# The neuroprotective effects of fisetin, a natural flavonoid in neurodegenerative diseases: Focus on the role of oxidative stress

**DOI:** 10.3389/fphar.2022.1015835

**Published:** 2022-10-10

**Authors:** Syed Shams ul Hassan, Saptadip Samanta, Raju Dash, Tomasz M. Karpiński, Emran Habibi, Abdul Sadiq, Amirhossein Ahmadi, Simona Bungau

**Affiliations:** ^1^ Shanghai Key Laboratory for Molecular Engineering of Chiral Drugs, School of Pharmacy, Shanghai Jiao Tong University, Shanghai, China; ^2^ Department of Natural Product Chemistry, School of Pharmacy, Shanghai Jiao Tong University, Shanghai, China; ^3^ Department of Physiology, Midnapore College, Midnapore, West Bengal, India; ^4^ Department of Anatomy, Dongguk University College of Medicine, Gyeongju, South Korea; ^5^ Department of Medical Microbiology, Poznań University of Medical Sciences, Poznań, Poland; ^6^ Department of Pharmacognosy, Faculty of Pharmacy, Mazandaran University of Medical Sciences, Sari, Iran; ^7^ Department of Pharmacy, University of Malakand, Chakdara, Pakistan; ^8^ Pharmaceutical Sciences Research Centre, Faculty of Pharmacy, Mazandaran University of Medical Sciences, Sari, Iran; ^9^ Department of Pharmacy, Faculty of Medicine and Pharmacy, University of Oradea, Oradea, Romania

**Keywords:** neurodegeneration, flavonoid, antioxidant, fiestin, oxidative stress

## Abstract

Oxidative stress (OS) disrupts the chemical integrity of macromolecules and increases the risk of neurodegenerative diseases. Fisetin is a flavonoid that exhibits potent antioxidant properties and protects the cells against OS. We have viewed the NCBI database, PubMed, Science Direct (Elsevier), Springer-Nature, ResearchGate, and Google Scholar databases to search and collect relevant articles during the preparation of this review. The search keywords are OS, neurodegenerative diseases, fisetin, etc. High level of ROS in the brain tissue decreases ATP levels, and mitochondrial membrane potential and induces lipid peroxidation, chronic inflammation, DNA damage, and apoptosis. The subsequent results are various neuronal diseases. Fisetin is a polyphenolic compound, commonly present in dietary ingredients. The antioxidant properties of this flavonoid diminish oxidative stress, ROS production, neurotoxicity, neuro-inflammation, and neurological disorders. Moreover, it maintains the redox profiles, and mitochondrial functions and inhibits NO production. At the molecular level, fisetin regulates the activity of PI3K/Akt, Nrf2, NF-κB, protein kinase C, and MAPK pathways to prevent OS, inflammatory response, and cytotoxicity. The antioxidant properties of fisetin protect the neural cells from inflammation and apoptotic degeneration. Thus, it can be used in the prevention of neurodegenerative disorders.

## Graphical Abstract

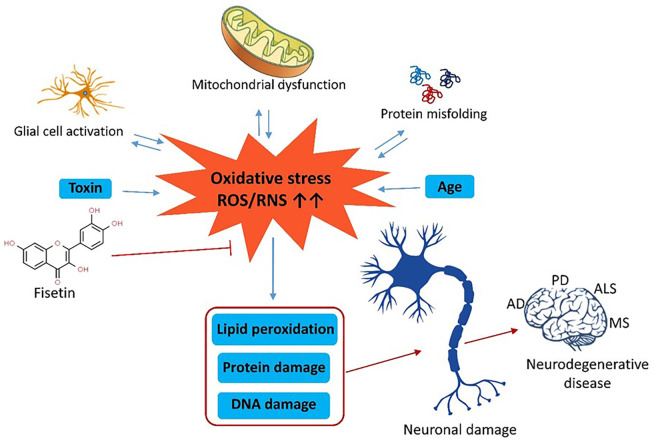

## Introduction

Neurodegenerative diseases are characterized by the permanent loss of a specific type of neurons in the brain, leading to progressive loss of brain functions. The subcortical area of the brain particularly basal ganglia is mostly affected in certain neurodegenerative diseases. Elderly people are vulnerable to neurodegenerative diseases (Wyss-Coray, 2016; Reddy et al., 2021). The most common neurodegenerative disorders are Alzheimer’s disease (AD), Parkinson’s disease (PD), Huntington’s disease (HD), and amyotrophic lateral sclerosis (ALS) have no specific treatment or cure. The common symptoms of these diseases are memory and cognitive impairments, and difficulty to move and speak (Gitler et al., 2017; Rong et al., 2020). Accumulation of specific proteins starts neurodegeneration. These include α-synuclein in PD, β-amyloid plaque and tau in AD, huntingtin in HD, and inclusion of transactivation response (TAR) DNA-binding protein (TDP)-43, superoxide dismutase 1 (SOD1), and FUS in ALS. The progressive loss of neuronal dysfunctions and death during neurodegenerative diseases are associated with proteotoxic stress, impaired activity of ubiquitin–proteasomal and autophagosomal/lysosomal systems, mitochondrial dysfunction, glutamate toxicity, calcium load, neuroinflammation, and aging (Behl et al., 2020; Karthika et al., 2022; Kaur et al., 2022).

Mitochondrial dysfunction and oxidative stress play crucial roles in the development of neurodegenerative diseases. Mitochondria are the main site for metabolic reactions where electrons are finally transported through the electron transport chain (ETC). Any impairment in mitochondrial function increases the production of reactive oxygen species like superoxide anion radicals (O−2) (Van Houten et al., 2006). Mitochondrial superoxide dismutase converts O−2 to hydrogen peroxide (H2O2) which is dissociated to water by the action of glutathione peroxidase (GPx). Excess production of ROS causes loss of ATP, decrease in the mitochondrial membrane potential, induction of apoptosis, and subsequent cell loss (Ott et al., 2007; Shams ul Hassan et al., 2021). ROS also induces lipid peroxidation, formation of the protein carbonyl compounds, and DNA damage. Thus, oxidative damage directly or indirectly advances AD, PD, HD, and ALS (McLennan and Esposti, 2000). However, reduced glutathione, vitamin E, and vitamin C can protect the cells by scavenging ROS (Sheikh et al., 2013).

Apoptosis and ferroptosis of neurons is an important features in neurodegenerative disorders. Human postmortem brain samples showed detectable DNA fragmentation in PD, AD, HD, and ALS. Moreover, the presence of caspases and BclXL is also associated with Alzheimer’s disease. Different caspases are involved in apoptosis. Caspase-6 plays an important role in the pathogenesis of AD and HD, whereas caspase-7 is activated during motor neuron degeneration in a mouse model of ALS. Thus, apoptosis causes chronic neurodegeneration and neuron loss. Abnormal expression of cell cycle regulator proteins has also been implicated in neurodegenerative diseases. Additionally, increased levels of p53 in motor neurons of the spinal cord and motor cortex induce neuronal degeneration (Bano et al., 2022).

**FIGURE 1:**
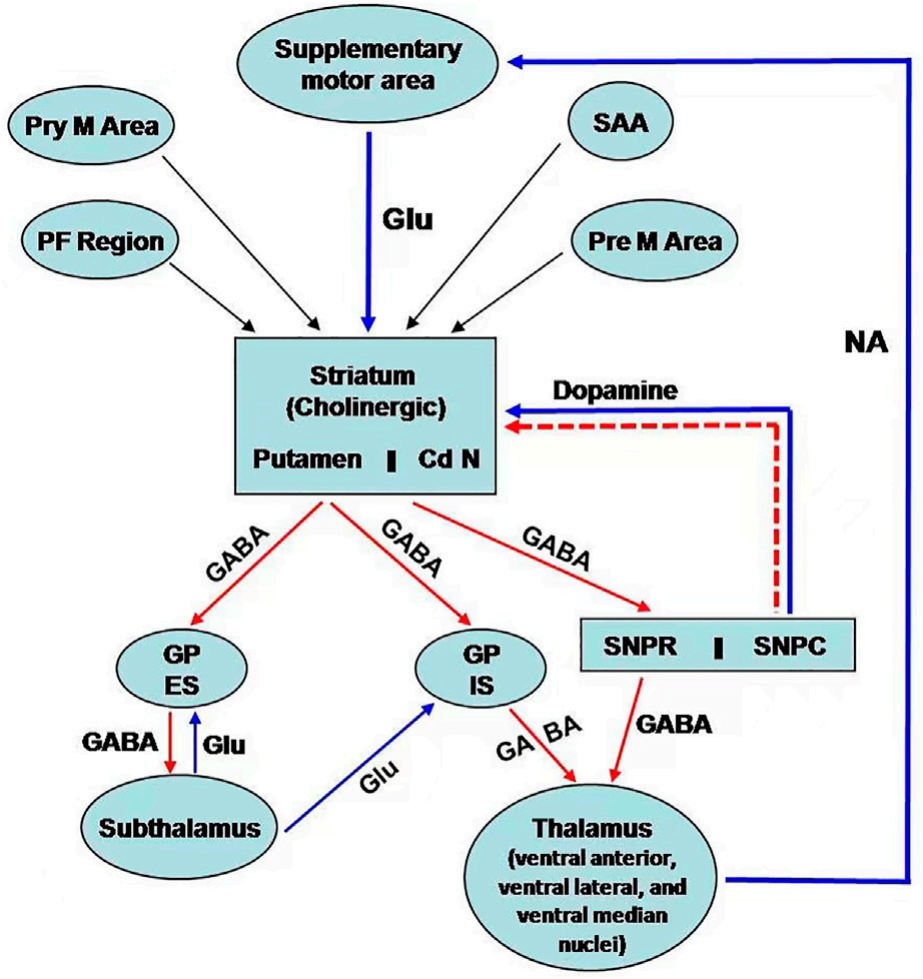
Schematic representation of neural connection of the basal ganglia. There are three main pathways: i) nigrostriatal dopaminergic pathway; ii) intrastriatal cholinergic pathway; iii) GABAergic pathway from the striatum to GP and SN. [PF cortex: Prefrontal; Pre M; Premotor; Pry M: Primary motor; SAA:Sensory associated area; Cd N: Caudate nucleus; GP ES: Globus Pallidus external segment; GP IS: Globus pallidus internal segment; SNPR: Substantia nigra pars reticularis; SNPC: Substantia nigra pars compacta; GLU: Glutamatre; GABA: Gama amino butyric acid; NA:Noradrenaline].

Basal ganglia are situated bilaterally in the white matter of the cerebral hemispheres and comprise five subcortical nuclei. These include the caudate nucleus, putamen, subthalamic nucleus, substantia nigra (SN), and the globus pallidus (GP). SN has two parts: 1) SN pars compacta and 2) SN reticularis/reticulata. On the other hand, GP has two segments: 1) GP internal and 2) GP external (Figure 1). The caudate nucleus, the putamen is collectively called striatum due to the striated appearance of these nuclei. The passing of the fiber bundle from the anterior limb of the internal capsule causes the formation of striations. Putamen and GP are known as the lenticular nucleus. The excitatory glutamatergic cortico-striate projections carry the impulse to the striatum from the cerebral cortex (Ehrlich, 2012). The basal ganglia also receive impulses from centromedian nuclei of the thalamus.

The efferent connections from basal ganglia terminate to the prefrontal and premotor cortex and the ventral lateral, ventral anterior, and centromedian nuclei of the thalamus via the thalamic fasciculus. SN projects connections to the other areas of the thalamus. Additionally, basal ganglia are associated with other nuclei in the diencephalons and mid-brain. Basal ganglia are mainly involved in the regulation of activity of the motor area of the cerebral cortex for controlling movements. This regulatory function occurs through the way of the thalamus. Basal ganglia also control cognitive functions.

The neurons of the pars compacta of SN are dopaminergic. The nigrostriatal pathway releases dopamine as a neurotransmitter in the striatum, particularly in the putamen. Dopamine acts as an excitatory neurotransmitter to modulate the activity of inhibitory pathways (GABAergic) from the striatum (specifically from putamen) to the internal segment of the GP (Figure 1) (Ehrlich, 2012). However, dopamine inhibits the activity of the projections from the striatum (from putamen) to the external segment of the GP, followed by inhibition to the subthalamic nuclei. This pathway decreases the activity of motor areas. The pleiotropic effects of dopamine are mediated by the presence of different receptors of dopamine. The D1 receptor is excitatory and associated with the activity of the internal segment of the GP, whereas the D2 receptor is inhibitory and regulates the function of the external segment of GP.

## Fisetin

### Chemistry

Fisetin, chemically 3,7,3′,4′-tetrahydroxyflavone or 7,3′,4′-flavon-3-ol is a polyhydroxy flavonoid with various pharmacological potentials like antioxidant (Khan et al., 2013), anti-inflammatory (Wu et al., 2022), anti-cancer (Lin et al., 2016), neuroprotective (Kumar et al., 2020) and is used in vascular dementia (Cordaro et al., 2022). It occurs in numerous fruits and vegetables, such as grapes, apples, onions, strawberries, and cucumbers (Cordaro et al., 2022). It is found in the range of 2–160 μg/g in different food plants, and the average daily intake of this flavonol in humans has estimated about 0.4 mg (Cordaro et al., 2022). Because of its various bioactive properties, fisetin is considered a health-promoting factor agent and some dietary supplements containing fisetin have been marketed (Grynkiewicz and Demchuk, 2019). Fisetin is a flavonol with diphenyl propane structure containing two aromatic rings linked via an oxygenated heterocyclic ring. It is supplemented with four OH group substitutions and one oxo group (Jash and Mondal, 2014). The bioactivity of fisetin mainly depends on the OH groups at 3, 7, 3′, 4′ positions, carbonyl group at four position and with the double bond between C2 and C3; this double bond (C2 = C3) and the OH group at C-7, as well as a OH group at C3’ in the ring B and at C3 are associated with the antioxidant activity of fisetin (Cos et al., 1998; Sengupta et al., 2004). Moreover, with the 4-keto and 7-OH groups at its core structure, fisetin has been shown to be able to reduce inflammation (Tordera et al., 1994). A possible quinone/quinine methide structure of fisetin has been suggested (Awad et al., 2001). More, as o-hydroquinone electrophilic compound, fisetin has been shown to act as a neuroprotective and antioxidant agent via activating the nuclear factor erythroid 2–related factor 2/antioxidant response element (Nrf2/ARE) pathway (Cordaro et al., 2022). Fisetin is naturally synthesized in various dietary plant resources and also acts as a coloring agent in plants (Prabhu and Bhute, 2012). It is also found in several trees and shrubs in Fabaceae, Anacardiaceae and Cupressaceae families (Pal et al., 2016). Despite its potent bioactivity, fisetin has low solubility in water (< 1 mg/ml) which leads to low absorption from the gut (Guzzo et al., 2006). Some studies have shown that co-crystallization of fisetin with caffeine, isonicotinamide and nicotinamide could improve the solubility and oral bioavailability of fisetin (Sowa et al., 2013; Sowa et al., 2014). Furthermore, complexation with cyclodexterins and nanoencapsulation (Sechi et al., 2016; Kadari et al., 2017; Cai et al., 2022) has been reported to increase the solubility and pharmacological activities of fisetin.

### Pharmacokinetics

The pharmacokinetics of fisetin has been investigated in several studies. It has been reported that after intraperitoneal administration of fisetin (223 mg/kg) in mice, its maximum plasma concentration was 2.5 μg/ml at 15 min and it was decreased biphasically with a half-life of 0.09 h; three metabolites of fisetin were detected in this study including two glucuronides and a methoxylated metabolite as 3,4′,7-trihydroxy-3′-methoxyflavone (geraldol) (Touil et al., 2011). Geraldol was also shown to be the dominant metabolite of fisetin in mice; it has been indicated that fisetin was quickly transformed into geraldol after administration at doses of 2 mg/kg (i.v.) and 100 and 200 mg/kg (p.o.) in mice (Jo et al., 2016). The oral bioavailability of fisetin was reported 7.8 and 31.7% for oral doses of 100 and 200 mg/kg, respectively. It was also shown that the Cmax and AUC values for geraldol were more than those of fisetin (Jo et al., 2016). Fisetin and its phase II conjugated forms have been reported to be partly excreted in biliary excretion through P-glycoprotein in rats (Huang et al., 2018) which was assumed due to its 3-OH group and a 2,3-double bond, resulting in high binding affinity to P- glycoprotein (Huang et al., 2018). Fisetin has been shown to be absorbed rapidly and extensively conjugated via sulfation and glucuronidation in mice. In addition, the highest tissue levels of fisetin in mice were in kidney, intestine and liver, respectively (Shia et al., 2009). Similarly, BBB permeability of orally administered fisetin has been reported in several studies (Maher et al., 2006; Maher, 2009; Krasieva et al., 2015). Fisetin exhibited a high brain uptake potential *in vitro* (Maher, 2009); also it was shown to disperse into the brain parenchyma after oral administration in mice (Krasieva et al., 2015). More, in addition to its BBB permeability, fisetin can also affect hippocampal synaptic plasticity indirectly through the peripheral system (He et al., 2018). As a major concern with the use of phytonutrients, their poor absorption and bioavailability remain a challenge in the final purpose of developing them for human use. A fully studied pharmacokinetic profile will be critical in determining the potential use in human (Rein et al., 2013).

### Safety and toxicity

Generally, due to their extensive distribution in dietary plants, flavonoids are supposed to be safe and associated with little or no toxicity (Syed et al., 2016). Oral administration of fisetin in animal studies has been around 5–25 mg/kg, while its intraperitoneal doses range from 0.3 to 3 mg/kg dissolved by dimethyl sulfoxide (Horwitz and Rakel, 2018). In some clinical trials (clinicaltrials.gov) the oral dose of 20 mg/kg/day for two consecutive days and/or for two consecutive months are mostly used (Sari and Soysal, 2020). In the acute toxicity study of fisetin, there were no shown toxicity signs/symptoms like decreased body weight, diarrhea, respiratory distress, restlessness, contractions and coma (Prasath and Subramanian, 2011; Touil et al., 2011). Furthermore, it did not show signs of toxicity at doses up to 2 g/kg in an acute toxicity study with no toxicity in the histopathological analysis of the heart, lungs, kidneys, liver, stomach, intestines, spleen and reproductive organs (Currais et al., 2014). LD50 values of fisetin in mice were reported as 250 mg/kg (intraperitoneal), 1700 mg/kg (oral), 200 mg/kg (intravenous), and 400 mg/kg (subcutaneous) in different routes of administration (Guo and Feng, 2017).

## Fisetin and its pharmacological activities

**FIGURE 2:**
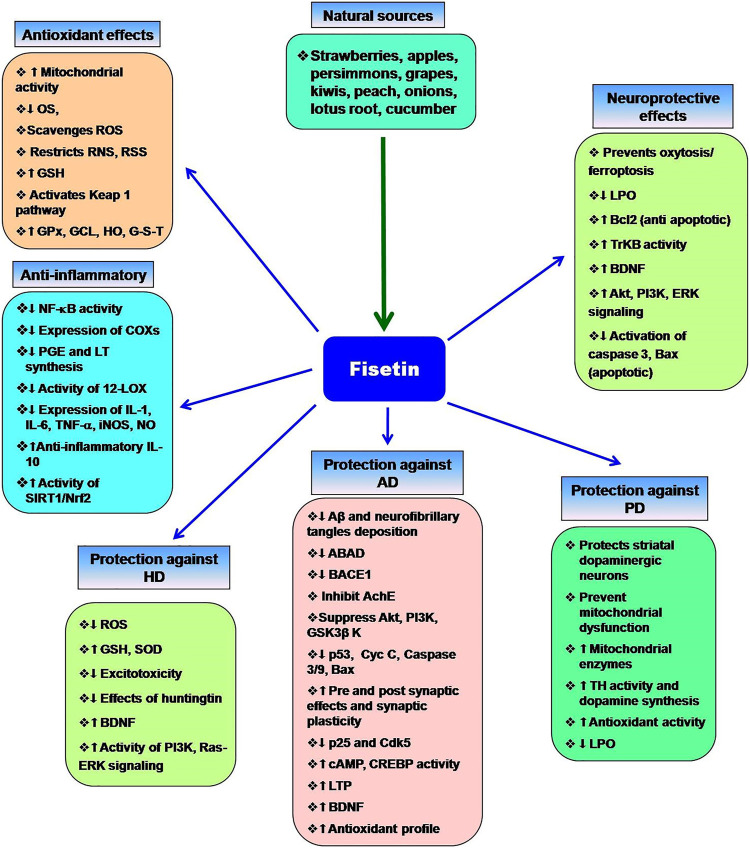
Multifaceted activities of fisetin and its neuroprotective functions. The role of OS the neurodegenerative diseases and the protective effects of fisetin.

Currently, some cholinesterase inhibitors and memantine are approved by Federal Drug Authority (FDA) as therapeutic agents to enhance the cognitive manifestations of AD without altering the progression of the disease pathology. In addition, research is being progressed to find the effective drugs to prevent its onset and progression (Kumar et al., 2015). Numerous natural products have extensively been shown to possess protective or therapeutic effects on neurodegenerative disorders via pharmacological activities, such as neurofunctional regulation, antioxidant, anti-apoptosis, anti-inflammatory, and calcium antagonization (Choudhary et al., 2013; Momtaz et al., 2020; Duan et al., 2022). Fisetin belongs to the class of flavonoids known as polyphenolic compounds. Flavonoids are pigments found in plants with their broad pharmacological spectrum as antioxidants, antiviral, anti-inflammatory, anticarcinogenic, antibacterial, neurotrophic, neuroprotective, and immune-stimulants (Reddy et al., 2021). Various fruits, including strawberries, persimmons, apples, grapes, kiwis, peach, onions, lotus root, and cucumber contain fisetin (2–160 μg/g) (Grynkiewicz and Demchuk, 2019; Hao et al., 2022). Several preclinical studies revealed that fisetin has potential benefits against neurological health complications and neurodegenerative diseases like AD, PD. HD, ALS, vascular dementia, schizophrenia, stroke, depression, diabetic neuropathy and traumatic brain injury. Fisetin acts as an antioxidant and anti-inflammatory agent (Figure 2) (Maher, 2020). Currais et al. (Currais et al., 2018), reported that fisetin decreases inflammation, senescent cells, and oxidative stress in rapidly aging SAMP 8 mice. Sagara et al. (Sagara et al., 2004) reported that fisetin was involved in nerve cell differentiation. Application of fisetin in rat embryonic origin nerve-growth factor (NGF)-expressing PC-12 cells showed neurite outgrowth and differentiation of PC-12 cells by activating the Ras-ERK pathway. Oxidative stress induces the depletion of the intracellular antioxidant reduced-glutathione (GSH) (Tan et al., 2001). This condition is very common in the aging brain (Currais and Maher, 2013). A vast body of evidence supports the ability of fisetin to serve as a protective mechanism against neurological diseases (Maher, 2015). Depletion of GSH increases mitochondrial dysfunction, ROS production lipoxygenase activation, lipid peroxidation and calcium influx, resulting in induction of cell death (Tan et al., 2001; Maher, 2019). Fisetin restores the mitochondrial activity, metabolic functions decrease oxidative stress, scavenges ROS. It exhibits direct antioxidant activity in addition to increasing intracellular antioxidants such as glutathione (Naeimi and Alizadeh, 2017). Additionally, fisetin has been indicated to regulate key neurotrophic factor-induced signaling pathways. It acts as a promising neuroprotective compound and prevents oxytosis/ferroptosis-mediated cell death (Ishige et al., 2001; Maher, 2019; Maher, 2020). Moreover, fisetin prevents lipid peroxidation and increases the antiapoptotic factor Bcl2 (Reddy et al., 2021). Fisetin prevents neuronal death from OS. It had been observed that fisetin protected HT-22 cells (derived from nerve cells of the central nervous system) and rat primary neurons against glutamate toxicity, hypoglycemia. This flavonoid increases reduced glutathione (GSH) and scavenges ROS, resulting in the prevention of (H2O2)-induced neuronal death (Ishige et al., 2001).

Chronic inflammation increases the risk of neuronal damage. Fisetin induces the expression of tropomyosin-related kinase B (TrkB), brain-derived-neurotropic factor, PI3K-Akt, and ERK signaling pathways and inhibits the synthesis of inflammatory agents during the treatment of depression (Yu et al., 2016; Uddin et al., 2020). Fisetin restores the proteasome activity in PD, AD, ALS (Maher, 2008). Fisetin improves neurodegenerative disease-associated dementia, cognitive functions and behavioral abnormalities along with increasing age (Maher, 2009; Currais et al., 2014; Maher, 2020; Reddy et al., 2021). Nuclear factor Kappa-B (NF-κB) is the key role player in the inflammatory pathway that induces the expression of several pro-inflammatory mediators, including interleukin-1 (IL-1), IL-2, IL-6, tumor necrosis factor- (TNF-α), and various enzymes like cyclooxygenase (COXs), inducible nitric oxide synthase (iNOS). These enzymes are essential for the synthesis of prostaglandin, nitric oxide (NO). Fisetin shows potent anti-inflammatory activity and prevents chronic inflammation (Gupta et al., 2016). It blocks the expression of cyclooxygenase enzymes, 12-lipoxygenase (12-LOX) and inhibits the synthesis of prostaglandin, and leukotriene (Wang et al., 2006). Fisetin prevents D-galactose-induced OS, neuroinflammation, and neuronal loss. It increases SIRT1/Nrf2, SNAP-25, and PSD-95. Alternatively, it decreases p-JNK/Nf-kB activity, activation of caspase-3, and the levels of Bax (Ahmad et al., 2021). Fisetin blocks the activity of Src, Syk, and IκBα and NF-κB, IKK, RIP, and TAK1 (Kim et al., 2015). Inhibition of Iκ-B restricts the phosphorylation of IKK, leading to inactivation of p65 subunit of NF-κB activity and the nuclear localization of NF-κB. Fisetin also blocks the overexpression of the p65 subunit. Fisetin-mediated inhibition of NF-κB activity suppresses the expression of inflammatory cytokines (Chen et al., 2018). Fisetin also increases the production of anti-inflammatory cytokine IL-10. Zhang et al. (Zhang et al., 2018) reported that fisetin facilitates the activity of nuclear factor erythroid 2-related factor 2 (Nrf2)-antioxidant response element (ARE) for neuroprotective functions. Fisetin induces the expression of Nrf2 with the help of activating transcription factor 4 (ATF4) gene, resulting in the elevation of GSH levels (Ehren and Maher, 2013). NF-κB interferes with the activity of Nrf2. In normal physiological conditions, a balance is maintained between the functions of NF-κB and Nrf2. This balance is disrupted as age progress. Fisetin inhibits the NF-κB activation and promotes Nrf2 activity to prevent neurodegeneration (Sivandzade et al., 2019). Kelch-like ECH-associated protein 1 (Keap 1)-Nrf2-ARE is the main regulator of redox signaling. This pathway regulates the expression of cytoprotective proteins, antioxidative enzymes (glutathione peroxidase, glutamate-cysteine ligase, heme oxygenase, NAD(P)H-quinine oxidoreductase and glutathione S transferase for regulating the redox homeostasis (Baird and Dinkova-Kostova, 2011; Jin et al., 2022). Under normal conditions, Keap1-mediated proteasomal degradation of Nrf2 suppresses the overexpression of antioxidant enzymes. However, oxidative stress releases Nrf2 from Keap1-complex that translocates to the nucleus and binds to ARE for the expression of antioxidant enzymes (Baird and Dinkova-Kostova, 2011; Sykiotis et al., 2011). Fisetin prevents the degradation of Nrf2 and increases its concentration, which advances the transcription of ARE-controlled antioxidant genes (Lee et al., 2011; Magesh et al., 2012; Reddy et al., 2021).

## Different neurodegenerative diseases and the protective role of fisetin

Neuroprotective effects of fisetin in different neurological disorders have been indicated in several studies. The reviewed studies have been discussed based on each disease including Alzheimer’s disease (AD), Parkinson’s disease (PD), Huntington’s disease (HD), Anxiety and depression, Amyotrophic Lateral Sclerosis (ALS), Multiple sclerosis, Age-related changes, Neuropsychiatric disorders, Vascular dementia and Cognitive dysfunction which are shown by detail in Table 1.

**TABLE 1:** Different neurodegenerative disease and the protective role of fisetin.

Disease	Cells*/in vitro*	Animals/*in vivo*	Dose/concentration/duration	Model/assay	Results	Ref
AD	-	-	Aβ1-42 was incubated for 0–48 h at 37°C	• Effect on Aβ fibril formation *in vitro*	• Inhibited Aβ fibril formation essentially via 3′,4′-dihydroxyl group	Akaishi et al. (2008)
AD	-	Mice	10 nM, 10 μM, or 1 mM Intraperitoneal injection (1.05, 2.1, or 3.15 mg/kg) (cromolyn sodium)	• *In Vitro* Aβ Fibrillization, oligomerization, and dissociation Assays • *In Vivo* Microdialysisinmice	• ↓Aβ Polymerization *in Vitro*	Hori et al. (2015)
					• ↓ The Concentration of Aβ40 in the interstitial fluid of APP/PS1 mice	
					• ↓ The half-life of Aβ within ISF	
					• ↓ The content of soluble Aβ *in Vivo*	
AD	Cultured rat hippocampal neurons	Mice	100 *μ*M; A*β*1-42 was incubated for 0, 2, 6 or 24 h A*β*1-42 was preincubated for 24 h with 10–100 *μ*M 3,3′,4′,5′-tetrahydroxyflavone added to hippocampal neuron cultures (dilution of 1:40)	• SDS-PAGE analysis and immunoblotting • Neurotoxicity assay with cultured rat hippocampal neurons	• Produced atypical, large A*β* aggregates	Ushikubo et al. (2012)
					• ↓ A*β* toxicity	
AD	-	Mice	15 mg/kg; P.O (14 days)	• Lipopolysaccharide and restraint stress-induced behavioral deficits model	• ↓ NF-κB, and IDO-1	Choubey et al. (2019)
					• ↑ Nrf-2, HO-1 and ChAT genes expression level	
					• ↓ AChE activity	
					• ↑ ChATactivity	
					• ↑ BDNF	
					• ↓ Augmentation of IL-1β	
					• ↓ Behavioral deficits	
AD	Mouse hippocampal HT22 cell BV2 microglial cells	Mice	25 and 50 µM 40 mg/kg; P.O	• LPS-stimulated BV2 microglia cells • Scopolamine-induced amnesia model	• ↓ Neuro-inflammation induced by LPS	Cho et al. (2013)
					• Inhibition of iNOS and COX-2	
					• ↑ The intracellular levels of GSH	
					• Restoring the declined activity of GSH-Px and SOD	
					• ↑ Memory	
					• Activation of the CREB–BDNF pathway	
AD	-	-	10–5 M 5, 20 and 50 × 10–6 M	• Determination of stabilizing effect on protein	• Stabilizing effect on protein • Protective effect against thermal denaturation	Giamogante et al. (2016)
AD	Mice	Mice	15 mg/kg; P.O; for 4 weeks before, and for 8 weeks during AlCl3-induction	• AlCl_3_-induced neuronal apoptosis and Aβ aggregation model	• ↓ Aβ aggregation, ASK-1, p-JNK, p53, cytochrome c, caspase-9 and 3 protein expressions	Prakash and Sudhandiran, (2015)
					• modulated Bax/Bcl-2 ratio	
					• ↓ TUNEL-positive and fluoro-jade C stained cells	
					• Regulating ASK-1 and p-JNK	
AD	-	-	100 nM; 24 h	• APP-Gal4 driven luciferase assay	• ↓ APP cleavage-dependent luciferase expression	Cox et al. (2014)
		Rats	10 and 50 mg/kg; P.O.; from gestational day 5 till parturition	• Developmental methyl mercury-induced neurotoxicity model	• ↓ Oxidative stress	Jacob and Thangarajan, (2017)
					• ↑ Enzymatic and non-enzymatic antioxidants	
					• ↑ The activity of membrane-bound ATPases	
					• ↑ Cholinergic function in F1 generation rats	
					• ↓ Morphological aberrations in brain	
AD	-	Rats	5, 10 and 25 mg/kg/day; P.O.; from the 14th day of L-methionine treatment for 2 weeks	• Hyperhomocysteinemia- induced vascular dementia	• ↑ SOD, CAT, GSH	Hemanth Kumar et al. (2017)
					• ↓ AChE, ↓LPO	
					• ↓ Behavioral deficits	
					• ↓ Histological aberrations	
AD	-	Mice	15 mg/kg; P.O.; 4 weeks before AlCl3 induction, or co-treatment 8 weeks	• AlCl3-induced neurotoxicity model	• ↑ SOD, CAT, GST, GSH	Prakash et al. (2013)
					• ↓ LPO, activity of AChE	
					• ↓ Behavioral deficits	
					• ↓ Histologic aberrations	
					• ↓ Reactive gliosis, and ↓ inflammatory niche	
AD	-	C57BL/6-Ins2Akita (Akita) mice	Fed in their food at 0.05%, resulting in a daily dose of approximately 25–40 mg/kg	• Ins2Akita model of type 1 diabetes	• ↓ Serum amyloid A • ↓ Anxiety-associated behavior	Maher et al. (2011b)
AD	-	Mice	25 mg/kg/day; P.O; for 3 months	• Rapidly aging senescence-accelerated prone 8 (SAMP8) mice model of sporadic AD and dementia	• ↓ Cognitive and locomotor deficits	Currais et al. (2018)
					• Restored the decrease of cytoskeleton-associated protein (Arc), Homer, and synapse-associated protein 102	
					• Altered changes in the levels of HSP40, HSP60, and HSP90	
					• ↓ The expression of the glial fibrillary acidic protein (GFAP)	
					• ↓ The activation of SAPK/JNK	
AD	Mouse cortical neuronal cells -Human neuronal H4 cell line -HEK293 cells	-	2.5–10 μM; 24 h 5–20 μM; 36 h 10 μM; 24 h	• Cell treatment with fisetin • Active GSK-3β-induced tau • aggregation model	• ↓ Phosphorylated tau	Kim et al. (2016)
					• ↓ Sarkosyl-insoluble tau	
					• ↑ Transcription factor EB ↑Nrf2 transcriptional factors	
					• ↑ Autophagy (↑LC3-II, beclin-1 and ATG7	
					• ↑ Number of autophagic vesicles in the cells)	
AD	Rat hippocampal slices	-	1µM; 5–20 min -0.1, 1, 5, 10 µM	• Cell treatment with fisetin	• ↑ ERK and ↑cAMP response element-binding protein (CREB) phosphorylation	Maher et al. (2006)
					• ↑ Long-term potentiation	
					• ↑ Object recognition in mice	
AD	-	-	6–200 µM	• BChE Enzyme inhibition assay	• ↓ The tested BChE usual, atypical, and fluoride resistant variants	Katalini et al. (2014)
AD	Primary rat neurons	-	10 µM	• SIN-1-mediated glutathione	• Protects against the alterations in ERK/c-Myc phosphorylation, nuclear Nrf2 levels, glutamate-cysteine ligase levels, GSH concentration and cell viability	Burdo et al. (2008)
AD	-	Rats	25 mg/kg/day; from the 36th to 62nd postpartum day of DL-Hcy treatment	• Homocysteine-induced chronic mild hyperhomocysteinemia model	• ↓ Behavioral deficits	Boyina et al. (2018)
					• ↑ Brain antioxidant (GSH, SOD, CAT) and nitrite levels	
					• ↓ Serum homocysteine,↑BDNF	
					• ↓ Pro-inflammatory biomarkers (TNF-α, IL-6)	
					• ↓ LPO	
					• ↓ Cholinergic and mitochondrial dysfunction	
					• ↓ Histological aberrations	
AD		Mice	25 mg/kg; P.O.; between the ages of 3 and 12 months in their food	• TheAPPswe/PS1dE9 double transgenic • AD mouse model	• Maintained learning and memory in AD mice	Currais et al. (2014)
					• ↑ ERK phosphorylation	
					• ↓ In protein carbonylation	
					• ↓ Oxidative stress	
					• ↓ p25	
					• ↓ Inflammation	
					• Alterations in global eicosanoid synthesis	
					• Maintenance of markers of synaptic function	
AD	-	Mice	20 mg/kg/day; i.p.; for 2 weeks starting 24 h after Aβ1–42 injection	• Aβ1–42 mouse model of AD • Aβ1–42-induced synaptic • dysfunction	• ↓ Synaptic dysfunction	Ahmad et al. (2017)
					• ↑ (SYN and SNAP-25)	
					• ↑ (PSD-95, SNAP-23, p-GluR1 (Ser 845), p-CREB (Ser 133) and p-CAMKII (Thr 286)	
					• ↑ Mouse memory	
					• Phosphorylation and activation of PI3K and phosphorylation of Akt (Ser 473) and GSK3β (Ser 9)	
					• Modulationg PI3K/Akt/GSK3β	
AD	HT22 hippocampal nerve cells MC65 human nerve cells BV-2 mouse microglial cells Rat PC12 cells	-	1–100 μM 10 μM 5 μM	• Oxidative glutamate toxicity assay • Anti-inflammatory activity (BV-2 cells) assay • Iodoacetic acid (IAA) induced *in vitro* ischemia • Intracellular amyloid toxicity (MC65 cells)	• Protective in oxytosis energy loss, ↓energy depletion	Fischer et al. (2019)
					• Antioxidant activity	
					• ↓ Inflammation	
					• ↓ Intracellular amyloid toxicity	
					• ↓ Inflammation mediated by microglial activation	
AD	-	Rats	25 mg/kgP.O.	• High-frequency stimulation-induced LTP	• Facilitated the induction of hippocampal LTP	He et al. (2018)
					• crossed the blood brain barrier	
					• ↑ Synaptic functions in the hippocampus	
AD	-	Mice	25 mg/kg; for 14 days; i.p	• LPS-Induced oxidative stress-mediated neurodegeneration and memory impairment	• ↓ ROS/oxidative stress	Ahmad et al. (2019)
					• ↑ p-JNK in hippocampus	
					• ↓ Gliosis	
					• ↓ TLR4/CD14/NF-kB signaling	
					• ↓ TNF-α, IL1-β, and COX-2	
					• ↓ Apoptotic neurodegeneration	
					• ↑ Hippocampal-dependent synaptic and memory functions	
AD	Primary microglia -BV-2 cells	-	0.5–2 μM	• Lipopolysaccharide-induced microglialactivation and neurotoxicity	• ↓ Production of TNF-α, NO, and prostaglandin (PG) E2	Zheng et al. (2008)
					• ↓ Gene expression of TNF-α, interleukin (IL)-1β, COX-2	
					• ↓ IκB degradation, nuclear translocation of NF-κB, and phosphorylation MAPKs	
					• ↓ Cytotoxicity in neuroblastoma cells	
Depression	-	Mice	5, 10, 25 mg/kg; i.v.; for the next 21 days)	• Reserpine-induced fibromyalgia model	• ↑ DA, NA and 5-HT	Yao et al. (2020)
					• ↑ Thalamic SOD and GSH	
					• ↓ MDA and NO	
					• ↑ The number of 5-HT positive neurons	
Depression	Mouse hippocampal HT22 nerve cells	Mice	10 μM(*in vitro*) 25 mg/kg; i.p.; P.O. (*in vivo*)		• Fisetin localized to the cell nucleoli	Krasieva et al. (2015)
					• Fisetin is rapidly distributed to the blood vessels of the brain with slower distribution into the brain parenchyma	
Depression	HT22 hippocampal nerve cells Mouse N9 microglial cells -PC12 cells	-	1–100 μM 10 μM 5 μM	• Oxidative glutamate toxicity assay	• Protective in oxytosis and *in vitro* ischemia assay, antioxidant activity • ↓ NO production	Maher and Kontoghiorghes, (2015)
				• Iodoacetic acid (IAA) induced *invitro* ischemia		
				• Neurite outgrowth in rat PC12 cells		
				• Enzymatic measurement of total glutathione (GSH)		
				• Intracellular ATP Assay		
				• Reactive oxygen species measurement		
				• LPS-stimulated NO production		
Depression	-	Mice	1–4 mg/kg, i.p.	• Oxaliplatin-induced neuropathic pain	• ↓ Depressive-like behaviour	Wang et al. (2015)
Depression	-	Adult male C57BL/6J mice	5, 15 or 45 mg/kg, p.o	• Chronic constriction injury model of neuropathic pain	• ↓ Monoamine oxidase (MAO) activity • ↑ Sevels of spinal monoamines • Modulating serotonergic system • ↓ Behavioural symptoms of depression and anxiety	Zhao et al. (2015a)
Depression	-	Abelson helper integration site-1 (Ahi1) knockout mice	5 mg/kg; i.p.; for 2 weeks	• Spatial restraint stress-induced depressive behaviours model	• ↓ Depressive phenotype • ↓ Restraint-induced increase in immobility • ↑ Phosphorylated TrkB • Activation of the TrkB signalling pathway	Wang et al. (2015)
Depression	-	Mice	10 and 20 mg/kg, p.o	• Reserpine-induced hypothermia and ptosis • p-chlorophenyl alanine (PCPA)-induced depletion of serotonin	• ↓ Immobility time in behavioural tests • The higher dose of fisetin: ↓ the hypothermia • ↑ Serotonin and noradrenaline levels in the frontal cortex and hippocampus • ↓ MAO • Regulation of the central serotonin and noradrenaline levels	Zhen et al. (2012)
Depression	-	Mice	20, 40 and 80 mg/kg; p.o.; for 7 days	• Lipopolysaccharide-induced • depressive-like behavior	• ↓ Alterations of the immobility time • ↓ IL-1β, IL-6 and TNF-α in the hippocampus and the prefrontal cortex • ↓ iNOS mRNA expression, ↓Nitrite levels via the modulation of NF-κB	Yu et al. (2016)
PD		Rotenone-induced PD in rat	Oral administration 10 mg/kg and 20 mg/kg, respectively	• Behavioral experiment through cylinder test to assess the motor functions • Estimation of TCA cycle enzymes, reduced glutathione, catalase, and MDA malondialdehyde (for LPO) • Estimation of striatal dopamine levels • Immunohistochemistry of substantia nigra for detection of tyrosine hydroxylase	• ↑Motor functions • Protect TCA cycle enzymes • ↑Striatal dopamine levels • ↑Glutathione, and catalase levels • ↓LPO • Restores tyrosine hydroxylase activity	Alikatte et al. (2021)
PD	MPTP/MPP toxicity in PC12		Doses range - 0.5–2 μg/ml	• Determination apoptosis through MTT assay and DNA fragmentation • Western blot and ELISA for detection of α-synuclein expression pro-inflammatory cytokine levels, and apoptotic markers	• ↓α-synuclein expression • ↓Inflammatory cytokines levels • ↓Apoptotic response	Patel et al. (2012)
PD	6-OHDA and paraquat toxicity in dopaminergic mouse MN9D cells		Doses range - 5–10 μM	• Determination oc cell survival	• ↑ Cell survival	Maher, (2017)
PD		MPTP- induced in mice model	10 and 25 mg/kg/bw	• Estimation of striatal dopamine levels • Immunohistochemistry study	• 25 mg/kg/bw is the effective dose • ↑Striatal dopamine level up to two folds • ↑ TH immunoreactivity in the dopaminergic nerves of SN	Maher, (2017)
PD		A case-control study in humans	Basic diet with good dietary sources of fisetin	• Clinical study of PD	• ↓ Symptoms of cogwheel rigidity, bradykinesia, dystonia, micrographia, hypomimia, constricted arm swing	Renoudet et al. (2012)
HD	PC12/HttQ103 cells expressing mutant Htt 1–103QP-EGFP		Pretreatment with ponasterone and supplementation of 2.5–10 lM fisetin	• Study of cell survival by MTT assay • Measurement of ERK and JNK activity through Western blot and densitometry • Estimation caspase by caspase 3/7 Glo kit	• ↑Cell survival by inducing Ras-ERK activation • Httex1-103QP-EGFP expression remains unaltered • ↓JNK phosphorylation and its activation • ↓ Caspase 3 activity	Maher et al. (2011a)
HD		R6/2 mice (mammalian model of (HD)	Dietary supplementation of fisetin (∼45 mg of/kg bw/day)	• Motor function test on the rotarod	• ↑Motor activity and survival • ↑30% life span • ↑ MAP-kinase activity and BDNF levels	Maher et al. (2011a)
ALS		hSOD1-G93A transgenic mice (B6SJL-Tg (SOD1-G93A) 1 Gur/J)	Oral supplementation of fisetin (9 mg/kg/day) via gavage	• Study of motor function by rotarod test • Immunohistochemistry study of spinal tissue	• ↓Motor deficits and ↑ motor functions • ↑Number of the motor neuron the spinal cord • ↓Mutant hSOD1	Wang et al. (2018)
ALS	hSOD1G93A NSC34 cells		Treatment of fisetin 10 μM	• Immunoblotting for detection of ERK, HO1, hSOD1 • Cell survival assay by cell counting kit 8 (CCK8) • DHE fluorescent probes for the study of ROS	• ↑Cell survival • ↓ROS levels • ↑Phosphorylated-ERK and HO1 activity • ↑ Expression of ERK and HO1	Wang et al. (2018)
ALS		*Drosophila* expressing mutant hSOD1G85R,	Oral supplementation of fisetin in different concentration (0, 1, 10, 100, or 300 μM) through food	• Immunofluorescence study detection of MAP2, HO1, hSOD1 • climbing activities assays • Estimation of GPx, catalase, and • Malondialdehyde (MDA) (for LPO)	• Higher concentration of fisetin improve motor ability • ↑Overall survival rate • ↑ CAT, GPx activity along with fisetin concentration • ↓MDA • ↑Phosphorylated-ERK • ↓ Mutant hSOD1	Wang et al. (2018)

NSC-34, cell line: Mouse neuroblastoma-embryonic spinal motor neuron fusion cell line; MAP2: Microtubule-associated protein 2; MDA: malondialdehyde; LPO: lipid peroxidation.

### Alzheimer’s disease

Alzheimer’s disease (AD) is a deadly progressive neurodegenerative disorder and the most common type of irreversible dementia (Reitz and Mayeux, 2014). Fundamentally, it is characterized by the presence of amyloid β 1–42 (Aβ) in extracellular neuritic plaques and intracellular neurofibrillary tau tangles (Goedert and Spillantini, 2006; Li et al., 2021; Li et al., 2022). Despite the Aβ deposition, several other molecular mechanisms (oxidative stress, neuroinflammation, hyperphosphorylated tau, intracellular signalling impairment, metal ion dysregulation, and familial association) are also implicated in AD (Webber et al., 2005). Aβ also increases the risk of excitotoxicity. Aβ generates free radicals and lipid peroxidation products that inhibit glutamate transporters to prevent the removal of excess glutamate from the extraneural space. The most affected neuronal subpopulations in AD are the cholinergic cells in the basal forebrain and the neurons of the hippocampus, cerebral cortex and the locus coeruleus. The expression of BDNF genes is reduced in Alzheimer’s patients that may be associated with a loss of cholinergic basal forebrain neurons (Sheikh et al., 2013). The common symptoms of AD are cognitive failure, visual-spatial confusion, memory, and personality. At the molecular level, c-Jun N-terminal kinase (JNK) is activated by different stimuli, including β-amyloid. Blocking of JNK signaling decreases the rate of apoptosis of neurons in response to β-amyloid. Thus, β-amyloid-induced JNK promotes Alzheimer’s disease. It is a known fact that the accumulation of Aβ peptide in the brain is the initial event in the AD process. More specifically, β-secretase (BACE1) and the γ-secretase complex cleave the amyloid precursor protein (APP), which leads to the production of Aβ species with different lengths (e.g., Aβ38, Aβ40, and Aβ42), wherein Aβ42 peptide is known to be more toxic (Liebsch et al., 2019). In this regard, a number of studies have been conducted on drugs that decrease Aβ production (such as inhibitors of BACE1), inhibit Aβ aggregation, or enhance brain Aβ clearance (Ayaz et al., 2019). Flavonoids are the major bioactive compounds that have a possible pharmacological role in neurodegeneration (Ayaz et al., 2019) Fisetin blocks the interaction between Aβ and a mitochondrial enzyme, amyloid-binding alcohol dehydrogenase (ABAD). Fisetin-mediated regulation of ABAD prevents mitochondrial dysfunction. It also inhibits the activity of β-site amyloid precursor protein cleaving enzyme 1 (BACE1) (Dash et al., 2014). Previously, Akaishi et al. (Akaishi et al., 2008) reported that fisetin inhibits Aβ aggregation and Aβ fibril formation. The results are low levels of amyloid production. Tau K-18 fibril formation decreases neuronal plasticity and promotes neurodegeneration. Fisetin inhibits tau-18 aggregation and neurofibrillary tangles formation (Xiao et al., 2021). Fisetin can also inhibit acetylcholinesterase (AChE) (Dash et al., 2014). Thus, fisetin counteracts AD. Flavonoids are one of the most studied natural compounds that are demonstrated to have a protective effect against neurodegenerative diseases like AD (Orhan et al., 2015; Liu et al., 2022). Fisetin, a class of flavonoids/polyphenolic compounds antagonizes the aggregation of Aβ by inhibiting its fibrillogenesis in *vitro* conditions. To investigate the structural requirements of fisetin for its anti-amyloidogenic activity, Aβ1-42 (20 µM) and several structurally related flavonoids were incubated, after that fibril formation was quantitatively determined. Fisetin was among the flavonoids that inhibited the formation of Aβ fibril; the 3′, 4′-dihydroxyl group was suggested to play an important role in its inhibitory effect (Akaishi et al., 2008). Although the C-3 OH group in flavonol does seem to play an essential role in the inhibition of fibril formation, the lack of an OH group at C-5 in fisetin in comparison to quercetin led to a significant difference in their inhibitory activity. A study conducted in the year 2014 showed that the inhibition of Aβ fibril formation by 3, 3′, 4′, 5′-tetrahydroxyflavone, a fisetin analogue, affects both Aβ conformation and neurotoxicity. Incubation of Aβ1-42 (20 μM) with 3, 3′, 4′, 5′-tetrahydroxyflavone (100 μM) was found to result in the formation of atypical Aβ conformers, distinguishable from soluble Aβ oligomers or mature Aβ fibrils in SDS-PAGE analysis. In addition, Aβ incubated with 3, 3′, 4′, 5′-tetrahydroxyflavone was shown to be less neurotoxic than Aβ without the flavonoid in cultured rat hippocampal neurons (Ushikubo et al., 2012). Some other compounds structurally similar to fisetin such as cromolyn sodium have also been shown to interfere with Aβ aggregation *in-vitro* and decrease the levels of soluble Aβ by over 50% in a transgenic model of Alzheimer’s disease in APP/PS1 mice (1 week; daily; i. p.) (Hori et al., 2015). Treatment with fisetin in Aβ-injected mice has also been indicated to reduce Aβ accumulation, tau phosphorylation, and correlated brain pathologies such as synaptic impairment, inflammation, and cell death. Administration of fisetin (20 mg/kg/day; i. p; for 2 weeks) 24 h after Aβ1–42 injection in mice, has been shown to significantly reduce the accumulation of Aβ, expression of BACE-1, and hyperphosphorylation of tau protein (Ahmad et al., 2017). Insoluble aggregates of neuronal tau proteins, called neurofibrillary tangles, are characterized as the histopathological hallmark of AD, which develops progressively in synaptic connections of the brain (Miao et al., 2019). *In vitro*, fisetin resulted in the reduction of phosphorylated tau in mouse cortical cells or primary neurons. It reduced the levels of sarkosyl-insoluble tau in an active GSK-3β-induced tau aggregation model (Kim et al., 2016). It is known that conditional elevated expression of GSK-3β activity in mouse hippocampal neurons results in cognitive impairment, hyperphosphorylation of tau, reactive microgliosis and astrogliosis, and neuronal death (Llorens-Martín et al., 2014). As downstream of PI3K, GSK-3β is known to be inactivated by Akt-mediated phosphorylation of serine residues of GSK3β (Zhang et al., 2014). In Aβ1–42-injected mice, fisetin improved memory and enhanced Aβ1–42-induced suppression of the PI3K/Akt/GSK3β signaling, which resulted in activation of p-PI3K, p-Akt (Ser473), and p-GSK3β (Ser9) expression. More, it suppressed various neuroinflammatory mediators and gliosis, and apoptotic neurodegeneration induced by Aβ1–42 in the mouse hippocampus (Ahmad et al., 2017). Furthermore, fisetin (15 mg/kg orally) treatment in mice, exhibited regulatory activity on Aβ aggregation and neuronal apoptosis induced by aluminium chloride (AlCl_3_). It significantly decreased Aβ aggregation and modulated the expressions of ASK-1, p-JNK, p53, cytochrome c, caspase-9 and 3 as well as Bax/Bcl-2 ratio in cortex and hippocampus of mice administered AlCl_3_. AlCl_3_-induced neurodegeneration in the cortex and hippocampus also decreased via fisetin as observed by TUNEL and fluoro-jade C staining (Prakash and Sudhandiran, 2015). Fisetin (100 nM) has been shown to inhibit APP-Gal4 dependent luciferase expression (67%) in a study evaluating various flavonoids for their probable modulatory effects on the APP-Gal4 driven luciferase assay (Cox et al., 2014). In mice experimental model, fisetin also reversed synaptic dysfunction induced by Aβ1–42, by increasing the levels of pre-and post-synaptic proteins, SYN and SNAP-25, PSD-95, SNAP-23, p-GluR1 (Ser 845), p-CREB (Ser 133) and p-CAMKII (Thr 286) (Ahmad et al., 2017). Currais et al., in 2017 reported that in a mice model for sporadic AD and dementia, fisetin decreased cognitive and locomotor deficits in old senescence-accelerated prone 8 (SAMP8) mice, along with restoring markers of impaired synaptic function and stress (Currais et al., 2018). It prevented a decrease of activity-regulated cytoskeleton-associated protein (Arc), Homer, and synapse-associated protein 102 (SAP102) in old mice compared to young mice; the expression of these proteins has previously been found to be decreased in old SAMP8 mice. Fisetin also altered the aging-induced changes in the levels of heat shock proteins (HSP40, HSP60, and HSP90) in hippocampal tissue. More, fisetin reduced the expression of the glial fibrillary acidic protein (GFAP) in the hippocampus of old SAMP8 mice, which was previously known as a marker for astrocyte activation. Moreover, fisetin prevented the activation of SAPK/JNK, as a marker of microglial activation in the old SAMP8 mice (Currais et al., 2018). Oral administration of fisetin to APPswe/PS1dE9 AD mice prevented the development of deficits in learning and memory through enhancing the phosphorylation of extracellular-signal related kinase (ERK). It also decreased the levels of p25 [the cyclin-dependent kinase 5 (Cdk5) activator p35 cleavage product] in AD mice. p25 overexpression has been known to be related to inflammation and astrogliosis and synaptic damage. More, fisetin exerted anti-inflammatory effects, including changes in eicosanoid synthesis as endogenous regulators of the inflammatory response. It affected the maintenance of markers of synaptic function in the AD mice (Currais et al., 2014). In a study in the year 2006, fisetin was shown to activate the signalling pathways in hippocampal slices involved in nerve cell survival and differentiation that are associated with the development of long-term memory. Fisetin treatment resulted in activation of cAMP response element-binding protein (CREB) through rapid stimulation of ERK phosphorylation in rat hippocampal slices; these effects were accounted for its ability to increase long-term potentiation (LTP) in hippocampal slices. It (in doses 10 and 25 mg/kg) also enhanced object recognition in mice, suggesting its use in memory disorders (Maher et al., 2006). Following, in 2017, Wen-bin He et al. showed that oral administration of fisetin (25 mg/kg) remarkably increased the induction of LTP at hippocampal CA1 synapses in rats and its effect was eliminated by intracerebroventricular injection of an agent with inhibitory effect in hippocampal slices, suggesting that oral fisetin crosses the blood brain-barrier (He et al., 2018). It has been shown that fisetin is rapidly distributed to the blood vessels of the brain after (intraperitoneal injection and oral) administration in mice, with slower distribution into the brain parenchyma. In mouse hippocampal HT22 nerve cells, fisetin has been shown to be localized to the nucleoli (Krasieva et al., 2015). In 2009, Burdo et al. indicated the peroxynitrite donor (SIN-1)-induced reduction in GSH levels and cell viability in primary rat neurons, which was through ERK/c-Myc phosphorylation pathway and a decrease in the expression of NF-E2-related factor-2 (Nrf2). These alterations in ERK/c-Myc phosphorylation, and the levels of nuclear Nrf2, and glutamate-cysteine ligase were shown to be restored by fisetin resulting in neuronal cell viability (Burdo et al., 2008). Fisetin treatment also resulted in autophagy induction and activation of transcription factor EB (TFEB) and Nrf2 transcriptional factors. It also decreased the phosphorylation levels of p70S6 kinase and 4E-BP1, as downstream proteins of mammalian target of rapamycin complex 1 (mTORC1), which shows that autophagy activating effect of fisetin might be through its mediating effect on mTORC1 inhibition (Kim et al., 2016). Cholinergic neurons are severely lost in AD (Mufson et al., 2008) and the cholinergic system plays role in several aspects related to AD such as learning, memory, synaptic transmission and neurogenesis (Ferreira-Vieira et al., 2016). The animal model of scopolamine-induced memory impairment is known as a well-established model based on acetylcholine metabolism (Yoon et al., 2018); memory deficit in this model is associated with the changed status of brain oxidative stress (Jeong et al., 2009). Fisetin isolated from the flavonoid-rich ethyl acetate extract of *Rhusverniciflua* bark has been indicated to decrease memory deficit induced by scopolamine in mice by the activation of the CREB–BDNF pathway and increase the intracellular GSH content by restoring the activity of GSH-Px and SOD (Cho et al., 2013). Its inhibitory effects against neuroinflammation also induced by LPS in BV2 microglia were observed through the inhibition of iNOS and COX-2. The extract of *Rhusverniciflua* bark also showed a protective effect on neurons against excitotoxicity and oxidative stress, via suppressing neuroinflammation *in vitro* (Cho et al., 2013). According to the co-regulatory effect of butyrylcholinesterase (BChE) in cholinergic neurotransmission, its inhibition appears to be of interest in AD. Fisetin is a flavonoid compound that has been shown to inhibit usual, atypical, and fluoride-resistant variants of human BChE with no significant change in potency in view of BChE polymorphism (Katalini et al., 2014). Mild hyperhomocysteinemia characterized by the increased amount of homocysteine in the blood is considered a risk factor for neurodegenerative diseases. In a rat model of chronic mild hyperhomocysteinemia induced by DL-homocysteine, fisetin was shown to restore the alterations in cognitive function, oxidative/nitrative stress, and some biochemical/morphological parameters in the brain cortex and hippocampus. Fisetin treatment prevented behavioral deficits, increased brain antioxidant, superoxide dismutase, catalase, reduced glutathione, and BDNF, consequently mitigating necrotic foci neurodegeneration in cortex and hippocampus. It also decreased serum homocysteine, and pro-inflammatory biomarkers (TNF-α, IL-6), lipid peroxidation, cholinergic dysfunction, mitochondrial dysregulation and decreased histological aberrations. A combination of fisetin and hesperidin showed better activity in neuroprotection (Boyina et al., 2018). Another study showed that fisetin in a dose-dependent manner, could attenuate L-methionine-induced behavioural deficits, and alterations in the levels of brain SOD, CAT, GSH, lipid peroxidation, and acetylcholinesterase activity in hyperhomocysteinemia (HHcy)-induced vascular dementia model in rats. It also decreased necrotic foci in the brain cortex (Hemanth Kumar et al., 2017). Fisetin administration (15 mg/kg; P.O) in mice model of LPS and restraint stress-induced behavioral deficits significantly ameliorated behavioural alterations. It also suppressed the up-regulation of NF-κB, and IDO-1 genes expression, and decreased the rise of IL-1β levels. The fisetin treatment also restored the downregulation of Nrf-2, HO-1, and ChAT genes expression and BDNF levels in the hippocampus, suggesting its protective effect against oxidative stress and neuro-inflammation as well as cholinergic dysfunction (Choubey et al., 2019). Moreover, in mice model of LPS-induced oxidative stress in the brain, fisetin pre- and co-treatment (20 mg/kg/day i. p) significantly abrogated the increased ROS/oxidative stress and activated phosphorylated c-JUN N-terminal Kinase (p-JNK) in the hippocampus; it also ameliorated LPS-induced activated gliosis, inflammatory Toll-like Receptors (TLR4)/cluster of differentiation 14 (CD14)/phospho-nuclear factor kappa (NF-kB) signalling, and TNF-α, IL1-β, as well as COX-2. Furthermore, results showed that fisetin significantly prevented LPS-induced apoptotic neurodegeneration and improved synaptic and memory functions dependent on the hippocampus in LPS-treated mice (Ahmad et al., 2019). *In vitro*, fisetin treatment in LPS-stimulated BV-2 microglia cells or primary microglia cultures, resulted in strong anti-inflammatory activity via suppression of TNF-α, NO, and prostaglandin E2 production, as well as inhibition of gene expression of TNF-α, IL-1β, COX-2 and iNOS at both mRNA and protein levels. It also inhibited IκB degradation, NF-κB, and phosphorylation of p38 mitogen-activated protein kinase (MAPKs) and decreased the cytotoxic effect of LPS-stimulated microglia on B35 neuroblastoma cells (Zheng et al., 2008). As a member of the protein disulphide isomerase family, PDIA3 is associated with various human pathologies such as cancers, Alzheimer’s, and Parkinson’s diseases. One study evaluated the interaction of some flavonoids with PDIA3 by quenching fluorescence analysis in which fisetin showed stabilizing effect on protein in a concentration-dependent manner and shared a protective effect against thermal denaturation (Giamogante et al., 2016). Administration of fisetin in pregnant rats resulted in protection against developmental neurotoxicity and behavioural impairment in weaning rats prenatally exposed to transplacental MeHg as a developmental neurotoxicity agent. Fisetin also reduced the levels of oxidative stress, enhanced the activity of membrane-bound ATPases, as well as cholinergic function in F1 generation rats and significantly prevented morphological aberrations in specific brain regions (Jacob and Thangarajan, 2017). In an *in vitro* study, fisetin, as a positive control, has been used to show the protection of nerve cells from cellular stress. It was effective at inducing neurite outgrowth in PC12 neuronal cells and could restore ATP and GSH levels respectively in the presence of IAA, and glutamate and prevent the glutamate-induced ROS generation (Maher and Kontoghiorghes, 2015). Fisetin also restored the AlCl_3_-induced reduction in the levels of SOD, CAT, GST, and GSH in a study that analysed the effect of this compound on AlCl_3_-induced reactive gliosis and neuronal inflammation in the brain of mice. Oral administration of fisetin in this model, altered the AlCl_3_-induced behavioural deficits, histologic aberrations, LPO, and compromised AChE activity (Prakash et al., 2013). In a study of the neuroprotective activity of the plant Yerba santa (*Eriodictyon californicum*), fisetin, as a positive control, exhibited protection in several assays reflecting multiple, age-associated neurotoxicity/survival mechanisms related to Alzheimer’s disease, such as elevated oxidative stress and GSH depletion, decreased energy metabolism, increase of aggregated proteins, inflammation and loss of neurotrophic support (Fischer et al., 2019). The actions of fisetin are summarized in Figure 2.

### Parkinson’s disease

Parkinson’s disease (PD) is a neurodegenerative disease (Kin et al., 2019), characterized by akinesia and bradykinesia as hypokinetic effects and rigidity and tremor, as the hyperkinetic effects. Difficulties occur in the initiation of motor movements, particularly the expression of unconscious movements like swinging of hands during walking, expression of facial movements related to emotion and others. Additionally, non-motor symptoms, such as gastrointestinal problems, sleep disorders, autonomic dysfunction, and sensory disturbances are also associated with PD (Schapira et al., 2017). This disease is caused by the deposition of alpha-synuclein and loss of dopamine secreting neurons in the pars compacta of SN (Michel et al., 2016). Loss of dopaminergic neurons reduces the excitatory activity of the striatum and increases the inhibitory activity of internal pathways and the output projections, resulting in suppression of functions of the motor cortex. Despite the dopaminergic neurons of SN, the loss of monoamine secreting neurons of the locus coeruleus and the raphe nucleus are also responsible for the development of PD. People of middle and old age are mostly affected by PD. It occurs in sporadic idiopathic form. The loss of dopaminergic neurons and dopamine receptors in the striatum are age-specific, which increases the progression of disease (Mandemakers et al., 2007). At the molecular level, three genes are associated with Parkinsonism. These genes encode the proteins parkin, ubiquitin carboxyterminal hydrolase L1 (UCH-L1) and α-synuclein. These proteins are involved in the removal of toxic proteins through a ubiquitin-dependent pathway. Impairment in this pathway increases the accumulation of toxic proteins that appears as the inclusion bodies such as Lewy bodies. The emergence of Lewy bodies is the well-known histological characteristic of Parkinsonism. Alpha-synuclein has been found in other neurodegenerative diseases like dementia, Lewy body dysphagia, pure autonomic failure, Shy–Drager syndrome, olivopontocerebellar atrophy and others (Karthika et al., 2022). This protein is one of the major constituents of Lewy bodies (Spillantini et al., 1997) and it is highly immunoreactive (Ishizawa et al., 2003). In addition to α-synuclein, β and γ members are also present. The number of amino acids varies from 127 to 140 in different synucleins. Alpha-synuclein may be involved in the trafficking of the synaptic vesicle (Südhof and Rizo, 2011). Fisetin interacts with α-synuclein and blocks its aggregation (Rane et al., 2021). Although there is no specific cause of PD; however, genetic analysis shows the hereditary relationship. The defect in chromosome 4q21–23 and the mutation in thr gene (A53T) encode a defective α-synuclein (Polymeropoulos et al., 1997). Alpha-synuclein binds with membrane lipid and undergoes a conformational change from amorphous to α-helical. Later, these helices are arranged in linear filaments, which pass through a structural rearrangement to form β-sheets that are similar to β-amyloid. This protein activates the JNK-dependent apoptotic pathway to promote the degeneration of dopaminergic neurons. JNK activates the apoptotic inducer AP-1 and promotes the expression of Fas-ligand for cell death. Moreover, the mutation in the α-synuclein gene (A53T) and synthesis of impaired α-synuclein increases the filamentous form of this protein that enhances the spreading of Lewy bodies. The immune reactive feature of Lewy bodies promotes mitochondrial damage that leads to progressive degeneration of nigrostriatal neurons. Dopamine secreting neurons of SN pars compacta exhibit a higher density of mitochondria that indicates the elevated rates of oxidative phosphorylation to meet the energy requirement (Pacelli et al., 2015; Liu Z. et al., 2022). The SNc neurons increase ATP production up to threefold and also promote ROS generation (Pacelli et al., 2015). A sufficient amount of energy is essential for the quality control of protein. Chaperones proteins can bind with unfolded/misfolded protein after hydrolysis of ATP. Thus, excess amount of energy is the supportive factor to prevent the accumulation of defective proteins in Lewy bodies. Another important cause is excitotoxicity. Glutamate is the stimulatory neurotransmitter in basal ganglia. One of its important receptors is N-methyl-D-aspartate (NMDA) receptor. An excess amount of glutamate activates NMDA receptors in SN pars compacta (Kure et al., 1991; Mattson and Magnus, 2006; Song and Wu, 2022). This binding withdraws magnesium-mediated blockage from the NMDA receptors that lead to stimulation of receptor, followed by intracellular calcium storm (Deister et al., 2009; Zweifel et al., 2009). During mitochondrial dysfunction and energy deficit conditions, intracellular calcium level remains high, resulting in oxidative stress and neurodegeneration in SN pars compacta (Blandini, 2001; Muddapu et al., 2019; Wang et al., 2021). The initial loss of dopaminergic neurons in SN diminishes the GABAergic inhibitory effect that aggravates excitotoxic degeneration processes (Muddapu et al., 2019). Inadequate calcium buffering increases the vulnerability of PD (Haddad and Nakamura, 2015). Brichta and Greengard (Brichta and Greengard, 2014) suggested that the presence of calbindin (intracellular calcium-binding protein) in dopaminergic neurons can use as a biomarker for PD. Although there is no specific neuroprotective treatment for PD (Kalia and Lang, 2015), fisetin exhibits protective effects against PD (Figure 2). The rotenone-induced PD in the rat model system had revealed that fisetin treatment improved motor function. This compound reverses the rotenone-induced changes in mitochondrial dysfunctions and protects mitochondrial enzymes (succinate dehydrogenase, citrate synthase, aconitase, and complex-I). Fisetin normalizes the activity of tyrosine hydroxylase (TH) and elevates striatal dopamine levels. It increases the concentrations of antioxidant components (glutathione, and catalase), and inhibits lipid peroxidation (Alikatte et al., 2021). Fisetin showed a significant positive impact on the protection of dopaminergic neurons in 1-methyl-4-phenyl-1, 2,3,6-tetrahydropyridine (MPTP)-treated PD in mice (Chen et al., 2020). Previously, Maher (Maher, 2017) observed that pre-treatment of fisetin before application of MPTP elevated dopamine level in the striatum of MPTP-induced mouse model of PD. He observed that the rate of dopamine synthesis is dose-dependent. Administration of 25 mg of fisetin/kg bw/day increased striatal dopamine level more than 2-fold, while 10 mg of fisetin/kg bw/day elevated the striatal dopamine level up to 70% from its basal level. MPTP also damages the tyrosine hydroxylase (TH) reactivity in dopaminergic nerve terminals of SN. Fisetin treatment protects the TH from its inactivation in the dopaminergic nerve terminals (Maher, 2017). Fisetin considerably facilitated the induction of long-term potentiation at rat hippocampal synapses that improved memory consolidation (He et al., 2018). A human case-control study had demonstrated that consumption of a diet rich in fisetin and hexacosanol for 6 months improved the clinical symptoms of PD such as cogwheel rigidity, bradykinesia, dystonia, micrographia, hypomimia, constricted arm swing with gait, and retropulsion (Renoudet et al., 2012). Fisetin activates the Ras-ERK pathway for the differentiation of norepinephrine and dopamine secreting PC12 cells (Ahmad et al., 2017). Wang et al. (Wang et al., 2019) reported that fisetin-like flavonoids regulated the activities of potassium channels and membrane potential that prevents Aβ_25-35_-induced neurodegeneration.

### Huntington’s disease (HD)

Huntington’s disease is a fatal neurodegenerative progressive disorder, where symptoms are disturbed psychiatric, cognitive and motor functions. The damage occurs in the spiny neurons of the caudate nucleus and putamen. Impaired activity of intrastriatal GABAergic and cholinergic pathways are the main cause of HD. The loss of inhibitory signal to the external segment of GP releases its inhibitory effect which ultimately increases the excitatory output signals from the thalamus, resulting in the hyperactivity of the motor cortex areas. The common symptoms are the jerky trajectory of the hands, hyperkinetic choreiform movement and slurred speech. Fundamentally, HD is an inherited autosomal dominant disorder. The gene responsible for the HD is present in the short arm of chromosome 4 (4p16.3). This gene is also called *HD*. Normally, this gene contains 11–34 (with a median of 19) glutamine-specific codon (CAG), but in the disease state this number increases up to 37–86 (with a median of 45) or more. The gene *HD* encodes a protein designated huntingtin, which is involved in the regulation of the transcription of neurotransmitter receptors in the brain. In the basal ganglia, huntingtin plays a vital role in transcriptional processes as well as axonal transport and membrane trafficking. The mutant form of huntingtin cannot regulate these functions and its accumulation promotes the risk of disease. Mutant huntingtin protein (Htt) is a misfolded soluble protein that contains a polyglutamine (poly Q) stretch. Htt-25Q is non-toxic, while Htt-103Q formed soluble aggregates and cytotoxicity (Xi et al., 2016). Increased levels of mitochondrial activity, formation of excess ATP as well as ROS are associated with HD. Impaired activity of ETC and high concentration of antioxidants had been shown in HD patients (Gu et al., 1996; Sorolla et al., 2008). Experimental evidence had proved that striatal cells produce ROS and reactive nitrogen species (RNS) (Ribeiro et al., 2013). Furthermore, oxidative and calcium-induced stress induces the striatal mitochondria for the production of inner mitochondrial transition pore (MTPs), resulting in induction of apoptosis (Brustovetsky et al., 2003). Glutamate-induced excitotoxicity increases intracellular calcium levels, which are initially buffered by calcium-binding proteins. Excess calcium decreases the expression of calcium-sensing proteins, hippocalcin thereby increasing the susceptibility of neuronal damage (Luthi-Carter et al., 2000; Maher, 2020). Moreover, Oxidative stress also increases huntingtin production even at normal glutamate levels (Estrada Sánchez et al., 2008). Zhao et al. (Zhao et al., 2011) reported that restoration of mitochondrial activity can prevent neuronal degeneration. Fisetin is a potent antioxidant. It is involved in quenching of ROS, restoration of GSH and SOD levels. It prevents mitochondrial dysfunctions and maintains the ATP levels in the cells. Collectively, these effects block the oxidative stress-induced excitotoxicity, followed by calcium inductive effects and synthesis of huntingtin (Figure 2). Experimental evidence indicated that expression of mutant huntingtin in PC12 cells resulted in the activation of multiple protein kinases, particularly mitogen-activated protein kinases (MAPKs), c-Jun N-terminal kinases (JNKs) and Ras-extracellular signal-regulated kinase (ERK) (Pal et al., 2016). These are mostly calcium-mediated effects. Fisetin regulates excitotoxicity-induced calcium release and the activation of kinases. Experimentally, fisetin treatment of PC12 cells expressing mutant huntingtin (Htt1-103QP-EGFP) increases Ras-ERK cascade and decreased phosphorylation-induced JNK activation, leading to suppression of caspase-3 activation and apoptosis, followed by increased cell survival (Maher et al., 2011a). *In vivo* experiment was conducted with transgenic R6/2 mice (a mammalian model of HD). R6/2 mice show aggressive disease phenotype and shortened lifespan. Feeding of fisetin-containing diet (∼45 mg of fisetin/kg bw/day) from the age of ∼6 weeks increased motor activity (tested on the rotarod from ∼7 weeks) compared to control group of R6/2 mice consuming fisetin-free diet. The rate of survival of R6/2 mice taking the fisetin-containing diet was ∼30% higher (139 days) compared to the control group (104 days) (Maher et al., 2011a). The effects of fisetin were also tested on *Drosophila* flies model expressing mutant human Httex1 in neuronal cells and exhibited HD-like symptoms. Feeding of fisetin-containing diet to *Drosophila* flies expressing pathogenic human Htt (w elav:Gal4/w; P{UAS-Httex1p Q93}/+) (Httex1p Q93) enhanced ERK phosphorylation and activation, resulting in ∼25% diminution of neurodegeneration, suppression of symptoms of HD and increased 77% of overall survival (Maher et al., 2011a). Fisetin mimics the activity of EGF-1 and BDNF. It can induce Ras-ERK signaling, followed by activation of MAP-kinase pathway that reduces the impact of mutant huntingtin (Strand et al., 2007; Maher et al., 2011a; Yan et al., 2020).

### Amyotrophic lateral sclerosis (ALS)

ALS is an age-related neurodegenerative disorder of motor neurons in the spinal cord, motor cortex and brain stem. The exact cause of ALS is still unclear. It has been assumed that glutamate-induced excitotoxicity is one of the possible causes of pathogenesis. Excess glutamate in the synapse or extracellular space is transported into neuronal and glial cells via glutamate transporter 1 (GLT-1) (Förstl and Kurz, 1999). Any mutation in GLT-1 increases glutamate-mediated excitotoxicity and also hampers calcium homeostasis. Glutamate can activate α-amino-3-hydroxy-5-methyl-4-isoxazole propionic acid (AMPA) receptors that influence greater calcium influx into the mitochondria, leading to oxidative stress. (Carriedo et al., 2000). ROS is another factor that promotes the occurrence of ALS. ALS patients carry mutations in the gene encoding superoxide dismutase (SOD1), resulting in poor ROS-scavenging capacity (Vielhaber et al., 2000; Menzies et al., 2002). Transgenic mice expressing the human SOD1 mutant variant (hSOD1-G93A) exhibited ALS. These animals did not have the effective antioxidant capacity and the rate of mitochondrial DNA damage was high (Menzies et al., 2002). Thus, oxidative stress and glutamate-induced cell death are associated with ALS. The antioxidant, anti-inflammatory, and anti-apoptotic effects of fisetin protect the motor neurons from degeneration. Oral supplementation of fisetin (9 mg/kg/day) in SOD1-G93A transgenic mice at the age of 2 months improves motor functions by delaying motor deficits. Additionally, fisetin treatment significantly increases the number of motor neurons in the spinal cord. This flavonoid also restricts the progression of ALS and increases survival (Wang et al., 2018). Fisetin modulates the phosphorylation of ERK and the expression of HO-1, GPx, and catalase for the reduction of oxidative stress (Wang et al., 2018). This flavonoid preserves mitochondrial SOD1 and resists mitochondrial DNA breakage. As a consequence, fisetin can fight against the development of ALS.

### Anxiety and depression

Depression and anxiety are considered as the psychological symptoms of stress which are highly comorbid disorders. These conditions are important factors upsetting mental health that affects the individual’s life. Depression is known as one of the most common psychiatric conditions and suicide risk factors, which results in the decrease of quality of life, increase in drug consumption, healthcare costs, and other social and economic problems (Whitney et al., 2019). Depression commonly occurs when the insufficient concentration of neurotransmitter (NT) is present in monoaminergic synapses. The imbalance of monoamine NTs is associated with depressive illness. Noradrenaline (NA) and 5-hydroxytryptamine (5-HT), as well as hypothalamic–pituitary–adrenal (HPA) axis are the most known involved systems in depression pathophysiology (Ferrari and Villa, 2017). Mutation in chromosome 11 causes defective expression of tyrosine hydroxylase, resulting in low levels of noradrenaline. The exogenous depressive agent like reserpine depletes monoamine NT and tends to increase depression. First-line treatments of depression and anxiety conditions are based on pharmacotherapy and cognitive behavioural therapy (de Abreu Costa et al., 2019). Monoamine oxidase (MAO) inhibitors such as clorgyline and deprenyline decrease the breakdown of monoamines and act as antidepressants. The reuptake of monoamine from the synaptic terminal is inhibited by reuptake inhibitors (imipramine and amitryptiline) that can improve depressive effects. Moreover, antidepressants downregulate the expression of presynaptic monoamine receptors. Fisetin shows an antidepressant effect. It acts as a monoamine oxidase (MAO) inhibitor and elevates serotonin and noradrenaline. Oral administration of fisetin to male ICR mice showed anti-depressant effects. This flavonoid inhibits monoamine oxidase activity to stop the degradation of neurotransmitters and elevates serotonin and noradrenalin production in the frontal cortex and hippocampus (Zhen et al., 2012). Stress response influences focal ischemia and glial activation that induces the expression of cytokines (TNF-α, IL-1β, and IL-6) for the advancement of depression. The stress response also activates NMDA receptors that transiently increase the expression of iNOS. The subsequent effect is the induction of cytokines. Experimentally, lipopolysaccharide (LPS) is the potent inducer of TNF-α expression. Fisetin regulates the levels of proinflammatory cytokines. It decreases the overexpression of iNOS TNF-α, IL-1β, and IL-6 in the hippocampus, and prefrontal cortex (Yu et al., 2016). Wang et al. (Wang et al., 2017) reported that fisetin activates the TrkB signal pathway through phosphorylation. This action exhibits the antidepressant effect.

Natural products are being used as alternatives to pharmacotherapy for the management of various diseases including depression and anxiety (Ferber et al., 2019). Among them, natural compounds such as flavonoids have been shown to decrease depressive symptoms in experimental models, possibly through the BDNF expression, monoaminergic systems as well as antioxidant effect (Hritcu et al., 2017). Evaluating the effect of fisetin on an LPS-induced depressive-like behaviour model in mice, showed that pre-treatment with fisetin (20, 40 and 80 mg/kg (orally); 7 days) could reverse LPS-induced increase in immobility time in forced swimming and tail suspension tests. In neurochemical assays, it showed inhibition of LPS-induced overexpression IL-1β, IL-6 and TNF-α in the hippocampus and the prefrontal cortex. More, in a higher dose, fisetin decreased the expression of iNOS mRNA and nitrite levels through modulation of NF-κB (Yu et al., 2016). In a rat model of reserpine-induced fibromyalgia, it was shown that fisetin treatment ameliorated reserpine-induced depression and inhibited the depletion of 5-hydroxytryptamine (5-HT), which was induced by reserpine in brain tissue. Fisetin also significantly inhibited the elevated oxido-nitrosative stress and ROS levels (Yao et al., 2020). In a mice model of chemotherapy (oxaliplatin)-induced neuropathic pain, fisetin has been shown to produce the anti-hyperalgesic effect in repeated treatment (not acute) and prevent chronic neuropathic pain-induced depressive-like behaviour in a dose-dependent manner. It was also shown that its effect might be mediated through serotoninergic 5-HT1A receptors as both antihyperalgesic and antidepressant-like effects were blocked by a selective 5-HT1A receptor antagonist (Wang et al., 2015). Another study showed that in an experimental model of neuropathic pain, fisetin decreased neuropathic hyperalgesia to thermal stimuli, and ameliorated the co-morbidly behavioural symptoms of depression and anxiety; mechanistically it was shown to modulate serotonergic system. Fisetin was indicated to increase the levels of spinal monoamines, and decrease monoamine oxidase (MAO) activity in chronic treatment (Zhao et al., 2015a). In one study, mice with depressive behaviour following spatial restraint exposure were administrated fisetin daily for 2 weeks and then evaluated for behavioural tests. In comparison with the control group, mice treated with fisetin did not show increased immobility time in the forced swimming and tail suspension tests. Furthermore, in Abelson helper integration site-1 (Ahi1) knockout mice, fisetin diminished the depressive phenotype. Mechanistically it was shown that fisetin might play its antidepressant effect by the activation of the TrkB signaling pathway which has a crucial role in the mechanisms of depression (Wang et al., 2017). Similarly, administration of fisetin (10 and 20 mg/kg, orally) in mouse models of despair tests leads to decreased immobility time, in the forced swimming and tail suspension tasks, and in a higher dose, fisetin reduced the hypothermia induced by reserpine. Its anti-immobility effect was eliminated by pre-treatment of the mouse with p-chlorophenyl alanine which induces serotonin depletion. In neurochemical assays, fisetin was shown to increase the levels of serotonin and noradrenaline in the frontal cortex and hippocampus. Treatment with fisetin inhibited monoamine oxidase (MAO) activity in the mouse brain by 14.7% with no affection on the activity of MAO-B (Zhen et al., 2012). Furthermore, fisetin exerted a protective effect against diabetes-associated anxiety behaviour in Akita mice which displayed decreased locomotor activity and increased time of immobilization, as indicative of anxiety behaviour. Akita mice fed fisetin, exhibited a remarkable decrease in the distance travelled and time ambulatory in the open field test (Maher et al., 2011b).

## Role of fisetin in other neurological disorders

*Multiple sclerosis:* Multiple sclerosis (MS) is a potential disease of the brain and spinal cord. It is a neuroinflammatory disease. The immune system attacks the myelin sheath on the neurons and causes permanent damage. The primary symptom is the inability to move. In MS demyelination starts when macrophages phagocytose myelin and produce inflammatory mediators. Fisetin inhibits *in vitro* myelin phagocytosis by decreasing the activity of macrophages. It potentially regulates the secretion of inflammatory mediators, chronic inflammation in the central nervous system and neurological deficits (Hendriks et al., 2003). Fisetin reduced ROS (superoxide, H_2_O_2_ and OH^•^) production without affecting the viability of macrophage cells. It also suppresses ROS-producing enzymes mainly xanthine oxidase, NADPH oxidase, etc. This plant derivative specifically decreases NF-κB-induced proinflammatory mediators like NO, IL-1, and TNF-α (Hendriks et al., 2003).

*Age-related changes:* Brain function declines with age and causes memory deficits including working memory, short-term memory spatial memory (Yankner et al., 2008). Age-related decline in brain function affects the normal activity of life. Fisetin protects age-related changes in the brain and increases memory functions. Supplementation of fisetin showed better results in the object recognition test (memory function test) in C57BL/6J male mice (Bevins and Besheer, 2006). Fisetin can improve long-term potentiation (LTP), but the effect is dose-dependent (He et al., 2018). The actions of fisetin are also pertinent in old rats, where the flavonoid improves the antioxidant capacity and decreases oxidative stress markers (Singh et al., 2018). Moreover, dietary supplementation of fisetin in old mice significantly declines age-related indicators of the brain (Yousefzadeh et al., 2018).

*Cognitive dysfunction*: Fisetin protects the brain against hyperhomocysteinemia-induced dementia and cognitive dysfunction. It increases brain antioxidant levels and reduces pro-inflammatory cytokines and serum homocysteine levels. Fisetin also increases BDNF activity to prevent neurodegeneration (Boyina et al., 2018).

*Vascular dementia*: Hyperhomocysteinopathy also causes vascular dementia. Fisetin-mediated regulation of serum homocysteine prevents endothelial dysfunction and increases NO availability and antioxidant enzymes. It also improves the capacity of spatial learning and working memory. Thus, supplementation is effective against vascular dementia (Kumar et al., 2017).

*Neuropsychiatric disorders*: Fisetin stimulates BDNF expression, leading to the improvement of behavior disorders (Bawari et al., 2019). Schizophrenia is a serious problem in certain cases. Fisetin is an active agent against schizophrenia and can be used for therapeutic purposes. It maintains hippocampal synaptic plasticity and memory functions. This flavonoid increases the surface expression of the GluA1 subunit of AMPA receptor. It also continues the phosphorylation of the AMPA receptors, as well as CaMKII, CREB, ERK1/2 (Zhan et al., 2021).

## The role of OS the neurodegenerative diseases and the protective effects of fisetin

Oxidative stress (OS) is the imbalance of production, accumulation, and detoxification of ROS/RNS inside cells and even in tissues (Pizzino et al., 2017; Naoi et al., 2019). The increased rates of oxidative stress that arise from increased production of ROS, reduction in the antioxidant response, or both, play a vital role in the progression of neurodegenerative diseases (Ahmad et al., 2019). On basis of observations OS is also portrayed as either an auto-propagating process means OS influenced excess ROS causes cellular damage and damaged molecules individually can act as ROS or become ROS (Salim, 2017). ROS is chemically reactive molecules which naturally generates during the cellular and molecular processes and takes part in cell survival, inflammation, and stressor response with the following diseases; cancer, NDD, allergy, muscle dysfunction, and cardiovascular disorders (Zuo et al., 2013; Zuo et al., 2015). ROS act as a useful secondary messenger in cell signaling at low concentrations. However, ROS may disrupt cellular macromolecules like DNA, proteins, and lipids at higher levels and long-term exposure, leading to apoptotic and necrotic cell death. (Chen et al., 2012). Oxidative stress in the neuronal microenvironment induces lipid, protein, and DNA oxidation and produces numerous by-products including alcohols, aldehydes, peroxides, cholesterol oxide, and ketones (Simonian and Coyle, 1996). Though ROS might not be the stimulus for neurodegenerative diseases, disease progression is likely to be accelerated by oxidative damage through interaction with mitochondria (Dias et al., 2013). It is noteworthy that neuronal cells are particularly susceptible to oxidative damage due to their high oxygen consumption, high polyunsaturated membrane acid content, and weak antioxidants defense (Rego and Oliveira, 2003). Mitochondrial dysfunction, associated with the aberrant development of ROS, is closely related to NDD (Albers and Beal, 2000). ROS is active in the brain and neurons and targets post mitotic cells, glial cells and neurons that are particularly susceptible to free radicals, causing neuronal damage (Gilgun-Sherki et al., 2001). Salganik reported that ROS also leads to apoptosis (Salganik, 2001). ROS can influence transcription factors (TF) that mediate cellular response against ROS (Patten et al., 2010). Excess ROS can also trigger antioxidant defense like Nrf-2. Nrf-2 activated through ROS and enhance the expression of GPX (glutathione peroxidase), PRX (Peroxiredoxins), SOD (superoxide dismutase), and HO (heme oxygenase) antioxidant enzymes (de Vries et al., 2008; Yang et al., 2022). Another TF NF-κB can also be activated by ROS (Patten et al., 2010). Moderate level of ROS can enable inactive NF-κB by removing its inhibitor, which gradually inhibits caspase depended on cell death and produces antiapoptotic proteins (Kriete and Mayo, 2009; Patten et al., 2010). ROS- driven OS plays a significant role in AD pathogenesis; particularly ROS leads to deposition of A*β* (Bonda et al., 2010). In AD patients, the excessive OS observed may be a consequence of N-methyl-D-aspartate-type glutamate (NMDAR) over activation. It was shown that the activation of NMDAR results in increased Ca2 + infiltration by stimulating cellular absorption and eventual generation of ROS/Reactive Nitrogen Species (RNS) (Nakamura and Lipton, 2010; Nakamura and Lipton, 2011). ROS plays a significant role in mediating JNK/stress-induced protein kinase signaling cascade. This pathway is correlated with Aβ-stimulated apoptosis and tau protein hyperphosphorylation (Patten et al., 2010). Moreover, A*β* proteins can induce ROS formation through the direct activation of NADPH oxidase (Nox) (Shelat et al., 2008). The pathway can also be modified via MAPK activation (Giraldo et al., 2014). The NADPH oxidase activity Increased OS resulting to enhance Aβ production can be triggered by aging, environmental stress, inflammation, and other nutrient factors such as redox-active metal ions (Aseervatham et al., 2013; Zeng et al., 2020). Moreover, OS can also be triggered by chemicals, pollutants, and radiation (Nizzari et al., 2012; Aseervatham et al., 2013). An excess amount of iron deposits also facilitates ROS formation (Nizzari et al., 2012). Oxidative stress can reduce α-secretase activity, promote β- and γ-secretase production and result in higher Aβ-production (Chen et al., 2008). OS stress also mediated the progression of HD. Recent studies indicated that OS decreases GLUT-3 expression, which inhibits cellular glucose transport (Reagan et al., 2000; Covarrubias-Pinto et al., 2015). It is also shown that in HD models mutated huntingtin (mHtt) protein inhibits normal mitochondrial functions, particularly impedes mitochondrial respiratory complex II functions (Lin and Beal, 2006; Bossy-Wetzel et al., 2008), which eventually increase ROS and decrease ATP (Bossy-Wetzel et al., 2008). In 2016 Liot et al. proposed that OS can inactivate GAPDH activity, which gradually leads to neuronal cell death (Liot et al., 2017). Pitts et al., demonstrated that development of mHtt protein reduced the activity of antioxidant protein peroxiredoxin Prx1 (Pitts et al., 2012). Moreover, OS modulate the aggregates conformation by increasing increase the size of mHtt (Mitomi et al., 2012). In PD, excess amount of ROS is produced due to mitochondrial dysfunction, neuroinflammation, dopamine degradation, aging, high levels of iron or Ca^2+^, and GSH depletion (Dias et al., 2013). Moreover, ROS accretion can be more severe due to pesticides, and other forms of neurotoxin interaction to PD individuals (Gangemi et al., 2016). Meiser et al. found dopamine causes the death of neurons in PD models (Meiser et al., 2013). Furthermore, dopamine-induced ROS causes proteasomal impairment, which subsequently causes neurodegeneration in PD (Ganguly et al., 2017). The rotenone and MPTP induced PD models demonstrated microglial NOX-2 activation, which eventually inhibits glucose transporter and ROS generation (Gao et al., 2003; Wu et al., 2003). It is evident that the lipid peroxidation biomarkers; 8-hydroxydeoxyguanosine (8-OHdG), 7β-and 27-hydroxycholesterol (7β/27HC), F2-isoprostanes (F2-IsoPs), 4-hydroxynonenal (4-HNE), 7-ketocholesterol (7-Kch), malondialdehyde, hydroxyeicosatetraenoic acid products (HETEs), and neuroprostanes (F4-NPs) increased in PD patients, which are supposed to be the cause of OS (Dalfó et al., 2005; Seet et al., 2010). About 20% of familial ALS occurs due to the mutation of the SOD1 gene (Gamez et al., 2006). SOD1 scavenges superoxide and maintains ROS (Saccon et al., 2013). The SOD1 mutants increase Nox2-dependent ROS production, which subsequently causes the death of neurons in ALS (Li et al., 2011). Interesting evidence has been shown that oxidized or misfolded wild SOD1 leads to mitochondrial dysfunction, which leads to the pathogenesis of ALS (D’Amico et al., 2013). Besides, by inhibiting neuroprotective pathway IGF-I/AKT, OS can cause neuronal cell death (Dávila and Torres-Aleman, 2008). The oxidative stress biomarkers such as 4HNE, thiobarbituric acid reactive substances (TBARS), 3-nitrotyrosine (3-NT), 8-OHdG, IsoPs, and advanced oxidation protein products (AOPP) increased in ALS patients (Oteiza et al., 1997; Smith et al., 1998; Bogdanov et al., 2000; Babu et al., 2008). In MS, OS plays a critical role. The inflammatory process of MS is most significantly mediated through OS. The activated macrophage and microglia generate a lot of free radicals, superoxide, hydroxyl radicals, nitric oxide and hydrogen peroxide. Furthermore, immature myeloid cells (MDSCs) also produce NO and ROS (Zhang et al., 2015; Ohl et al., 2016). Several studies demonstrated that, microglia produced ROS, oxidized DNA, lipids, and other mediators in MS (Haider et al., 2011; Fischer et al., 2013). Moreover, activation of effector T cells are also hampered by OS. Long term exposure to ROS reduces T cell proliferation which eventually induce programmed cell death (Kesarwani et al., 2013). There is a lot of evidence showed that, ROS leads to the production and progression of tissue damage in MS and stimulating the Nrf2 pathway may be a protecting mechanism in MS’ pathogenesis (Ohl et al., 2016). Some studies suggest that OS biomarkers such as 8-iso-PGF2*α,* AOPPs, IsoP, clusterin, isoprostanes, and 4-HNE increased in MS patients (Sbardella et al., 2013; Guan et al., 2015; Zhang et al., 2022).

**FIGURE 3:**
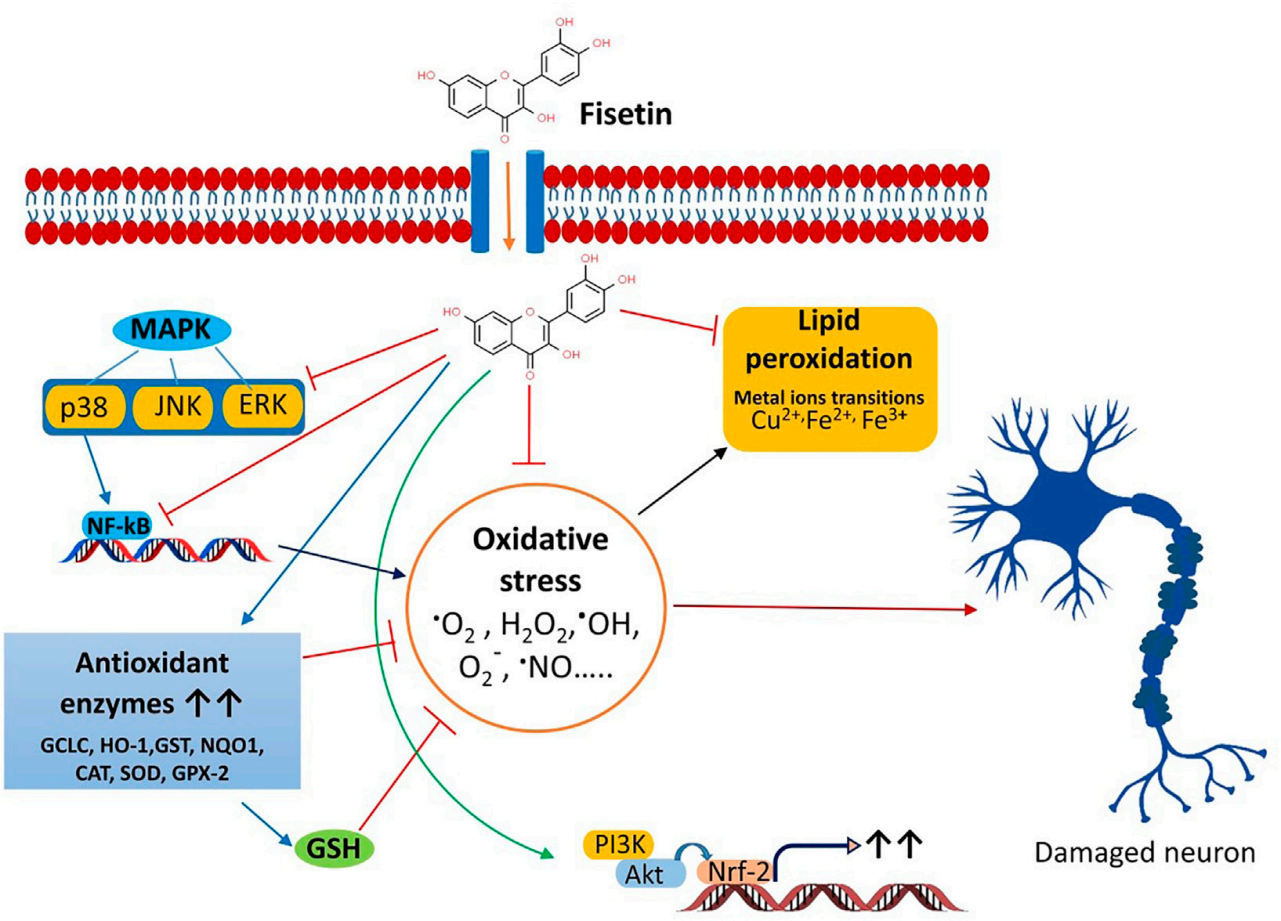
OS pathogenicity in neurodegenerative disease and the protevtive effects of fisetin.

A common dietary flavonoid fisetin, recently gained much scientific consideration for its antioxidant properties to inhibit a wide range of life-threating diseases (Zhang et al., 2019). Antioxidants diminish oxidative stress. Epidemiologically, several natural compounds (such as free radical scavenging) have been suggested as potential inhibitors which delayed oxidative reactions to mitigate or management of different NDD diseases (Sengupta et al., 2004). Fisetin is such a compound that can inhibit ROS and prolonged neuronal survival through different mechanisms (Figure 3) (Prakash and Sudhandiran, 2015; Ahmad et al., 2019; Zhang et al., 2019). The TEAC (Trolox-equivalent activity concentration) of fisetin was recorded to 2.80–0.06 (Ishige et al., 2001). An integrative study conducted by Currais et al., demonstrated that fisetin decreased neurological impairment in the adult SAMP8 mice by diminishing oxidative stress and neuroinflammation (Currais et al., 2018). Ahmed et al., indicated that fisetin is strongly neuroprotective against neurotoxicity caused by Aβ1-42 in AD mouse models (Ahmad et al., 2017). They also found that fisetin significantly reduce the level of ROS/oxidative stress induced by LPS (Ahmad et al., 2019). In another study Jhonsa et al., found that fisetin improved ROS homeostasis, levels of catalase, and superoxide dismutase (SOD) enzymes in PD *drosophila* (Jhonsa et al., 2016). Moreover, in a rat PD model, Alikatte et al., found that fisetin decreased rotenone-encouraged cognitive deficiencies, oxidative stress, and mitochondrial dysfunctions (Alikatte et al., 2021). Besides, in an aging rat model, fisetin reduced pro-oxidants substantially and boosted the antioxidant level (Baird and Dinkova-Kostova, 2011). Wang et al., also found that in ALS hSOD1 mutant models, fisetin therapy offered neuroprotection with enhanced neuronal survival, reduced motor dysfunction, decreased ROS level, and controlled redox homeostasis. In addition, fisetin enhanced phosphorylated ERK expression and upregulation of antioxidant molecules (Wang et al., 2018). Besides direct antioxidant activity, fisetin can also enhance glutathione (GSH), which is the most powerful intracellular antioxidant. GSH has significant role in redox homeostasis. This has been also proposed that fisetin can increase GSH levels either through enhancing the influx of cysteine and/or boosting the activity of GCL (glutamate cysteine ligase) (Maher, 2009). In a previous study, it was also found that fisetin was able to retain GCL levels decreased by peroxynitrite. The level of GCL’s expression is probably influenced by Nrf-2 (Burdo et al., 2008). In oxidative stress conditions, fisetin can also restore mitochondrial function. It also confers microglial cells anti-inflammatory effect and impedes the development of 5-lipoxygenase to minimize the formation of pro-inflammatory cytokines and lipid peroxide (Maher, 2009). The cellular GSH concentration are typically regulated by a complex series of mechanisms, including the availability and transportation of substrate, synthesizers and regeneration rates, utilization of GSH, and extracellular efflux (Meister and Anderson, 1983). In 2001 the neuroprotective effect of fisetin was first recognized against oxidative glutamate toxicity by Ishige et al. (Ishige et al., 2001). Kang et al., also found that fisetin decreased oxidizing agent superoxide, cell-free hydroxyl radical amount, and H_2_O_2_ induced intracellular ROS. Moreover, fisetin protected cells from lipid peroxidation, carbonylation of protein, and also DNA damage induced by H_2_O_2_ (Kang et al., 2014). In another study, Piao et al., found the same result where ROS was generated through γ-irradiation (Piao et al., 2013). Tingting Wang et al., found that fisetin protects DNA damage caused by ROS through hydrogen atom and/or single electron donation (HAT/SET) pathways (Wang et al., 2016). In addition, in an *in vitro* study, fisetin was reported to inhibit LDL oxidation (Myara et al., 1993). Chuang et al., 2014 et al., indicated that fisetin therapy effectively blocked LPS/IFN-γ induced nitric oxide (NO) and inducible nitric oxide synthase (iNOS) production in microglia. However, peptidoglycan stimulated iNOS and NO were also reduced by fisetin (Chuang et al., 2014). Recently Hussain et al., found that fisetin dramatically increased the expression of glutathione peroxidase-2 (GPX-2), SOD, hemoxinase-1 (HO-1), Nrf2 and decreased NO amount in CS‐induced rats (Hussain et al., 2019). Xu et al., suggested that fisetin nanoparticle has a significant effect of reducing oxidative stress of the NF-κB pathway on glial cells induced by PM2.5 (Xu et al., 2020). However, the details mechanism of action of fisetin is still not well understood. It is revealed that this high lipid-compound can efficiently be transported across lipid bi-layer, assembled within cells and exerts significant anti-inflammatory (Prakash et al., 2013), antioxidant activity (Prasath et al., 2013), and neuroprotective (Maher, 2009) activity both *in vivo* and *in vitro* models. It can also inhibit lipid peroxidation by preventing free radicals from further entering into the lipid center (Sinha et al., 2014). Fisetin can act together with various redox-related pathways such as PI3K/Akt, Nrf2, NF-κB, protein kinase C, tyrosine kinases, and MAPK once enters into the cell via different mechanisms, for example, metal ions chelation, acting as oxidoreductase substrate and enhancing non-enzymatic and enzymatic intracellular antioxidants (Mansuri et al., 2014; Naeimi and Alizadeh, 2017). In particularly, fisetin is able to develop Cu^2+^, Fe^2+^ and Fe^3+^ complexes which may also be stronger antioxidant effect than others (Kasprzak et al., 2015). Besides, several studies indicated that fisetin can chelates iron ions within a broad pH value compared to other flavonoids (3–10) (Dimitrić Marković et al., 2011; Naeimi and Alizadeh, 2017). Moreover, in the Aβ1-42-treated AD mice model, fisetin effectively triggered p-Akt (Ser 473), p-GSK3β (Ser 9), and p-PI3 K expression (Ahmad et al., 2017). Besides, fisetin also repressed P38 phosphorylation in microglial activation and neurotoxicity triggered by lipopolysaccharide (Zheng et al., 2008). Lower Nrf2 expression is related to oxidative stress. Nrf2 is a significant factor in redox homeostasis regulation and the synthesis of several cytoprotective molecules and antioxidants (Zhang et al., 2013). Sakai et al., found that *in vitro*, fisetin induced Nrf2 expression and also upregulated several oxidative stress-related enzymes like as GCLC, NADPH quinone oxidoreductase-1 (NQO1), HO-1, and Glutathione S-transferase (GST) and even phase II detoxifying enzymes (Sakai et al., 2013; Chuang et al., 2014; Naeimi and Alizadeh, 2017). Moreover, it is also proved in several animal models that inducing Nrf2 through fisetin could also enhance antioxidant production (Prasath and Subramanian, 2013; Zhao et al., 2015b; Zhang et al., 2021). Léotoing et al., found that IkB phosphorylation can also be inhibited by fisetin, which eventually blocks pro-oxidant genes expression and activation of NF-κB (Léotoing et al., 2013). P Maher also reported that fisetin could boost Nrf-2 levels in several neurons, such as retinal ganglion cells (Maher and Hanneken, 2005) HT22 cells (Maher, 2006), and primary cortical neurons (Maher, 2009). The fisetin also influenced to modulate Keap-1 to interact Nrf-2 to enhance the expression and concentration oxidative stress reduction related genes (Zhang et al., 2021). Nrf2 interacts with the antioxidant response element (ARE; EpRE, too), which controls the expression of several proteins associated with the prevention of oxidative stress and management of redox homeostasis of cells. (Nguyen et al., 2003; Chen and Kong, 2004; Zhuo et al., 2020). These conclusions agree with previous studies that showed fisetin may improve expression ARE-related genes in various non-neuronal cell lines (Hou et al., 2001; Myhrstad et al., 2002). So, it could be hypothesized that fisetin exerts neuroprotective roles targeting of several oxidative stress-related pathways components, including JNK, p38, ERK of MAPK, and also NF-κB or PI3K/Akt pathways (Naeimi and Alizadeh, 2017).

## Conclusion

Neurological disorders are a serious problem throughout the world and there is no definite treatment for this purpose. Excess production of ROS and poor detoxification process increase OS. Neurons are the most vulnerable to OS as they consume a greater amount of oxygen, contain much amount of polyunsaturated fatty acids in their membrane, and fail to maintain redox homeostasis. OS starts lipid peroxidation, protein carbonyl formation, DNA damage, induction of neuroinflammatory response, and apoptotic activity. Collectively, these responses promote the degeneration of neurons in the CNS. Fisetin belongs to flavonoid groups, scavenges ROS, and restricts OS. It also increases the expression of SOD, catalase, GPx, HO-1. Several studies indicate that fisetin is effective against AD, PD, HD, MS, and ALS. The multifaceted actions of fisetin increase the possibilities of its use to prevent neurodegenerative diseases. Finally, it can say that extensive research on the application of fisetin against CNS disorders and proper clinical trials will allow counteracting the neurological disorders.

## References

[B1] Ahmad A. Ali T. Park H. Y. Badshah H. Rehman S. U. Kim M. O. (2017). Neuroprotective effect of fisetin against amyloid-beta-induced cognitive/synaptic dysfunction, neuroinflammation, and neurodegeneration in adult mice. Mol. Neurobiol. 54 (3), 2269–2285. 10.1007/s12035-016-9795-4 26944285

[B2] Ahmad A. Ali T. Rehman S. U. Kim M. O. (2019). Phytomedicine-based potent antioxidant, fisetin protects CNS-insult LPS-induced oxidative stress-mediated neurodegeneration and memory impairment. J. Clin. Med. 8 (6), 850. 10.3390/jcm8060850 31207963 PMC6616651

[B3] Ahmad S. Khan A. Ali W. Jo M. H. Park J. Ikram M. (2021). Fisetin rescues the mice brains against D-Galactose-induced oxidative stress, neuroinflammation and memory impairment. Front. Pharmacol. 12 (57), 612078. 10.3389/fphar.2021.612078 33716741 PMC7947859

[B4] Akaishi T. Morimoto T. Shibao M. Watanabe S. Sakai-Kato K. Utsunomiya-Tate N. (2008). Structural requirements for the flavonoid fisetin in inhibiting fibril formation of amyloid beta protein. Neurosci. Lett. 444 (3), 280–285. 10.1016/j.neulet.2008.08.052 18761054

[B5] Albers D. S. Beal M. F. (2000). Mitochondrial dysfunction and oxidative stress in aging and neurodegenerative disease. J. Neural Transm. Suppl. 59, 133–154. 10.1007/978-3-7091-6781-6_16 10961426

[B6] Alikatte K. Palle S. Rajendra Kumar J. Pathakala N. (2021). Fisetin improved rotenone-induced behavioral deficits, oxidative changes, and mitochondrial dysfunctions in rat model of Parkinson’s disease. J. Diet. Suppl. 18 (1), 57–71. 10.1080/19390211.2019.1710646 31992104

[B7] Aseervatham G. S. B. Sivasudha T. Jeyadevi R. Arul Ananth D. (2013). Environmental factors and unhealthy lifestyle influence oxidative stress in humans—An overview. Environ. Sci. Pollut. Res. Int. 20 (7), 4356–4369. 10.1007/s11356-013-1748-0 23636598

[B8] Awad H. M. Boersma M. G. Boeren S. van Bladeren P. J. Vervoort J. Rietjens I. M. (2001). Structure–activity study on the quinone/quinone methide chemistry of flavonoids. Chem. Res. Toxicol. 14 (4), 398–408. 10.1021/tx000216e 11304128

[B9] Ayaz M. Sadiq A. Junaid M. Ullah F. Ovais M. Ullah I. (2019). Flavonoids as prospective neuroprotectants and their therapeutic propensity in aging associated neurological disorders. Front. Aging Neurosci. 11, 155. 10.3389/fnagi.2019.00155 31293414 PMC6606780

[B10] Babu G. N. Kumar A. Chandra R. Puri S. K. Singh R. L. Kalita J. (2008). Oxidant–antioxidant imbalance in the erythrocytes of sporadic amyotrophic lateral sclerosis patients correlates with the progression of disease. Neurochem. Int. 52 (6), 1284–1289. 10.1016/j.neuint.2008.01.009 18308427

[B11] Baird L. Dinkova-Kostova A. T. (2011). The cytoprotective role of the Keap1-Nrf2 pathway. Arch. Toxicol. 85 (4), 241–272. 10.1007/s00204-011-0674-5 21365312

[B12] Bano I. Horky P. Abbas S. Q. Majid M. Bilal A. H. M. Ali F. (2022). Ferroptosis: A New Road towards Cancer Management, 27. 10.3390/molecules27072129 Mol. (Basel, Switz. PMC9000380 35408533

[B13] Bawari S. Tewari D. Argüelles S. Sah A. N. Nabavi S. F. Xu S. (2019). Targeting BDNF signaling by natural products: Novel synaptic repair therapeutics for neurodegeneration and behavior disorders. Pharmacol. Res. 148, 104458. 10.1016/j.phrs.2019.104458 31546015

[B14] Behl T. Kaur G. Bungau S. Jhanji R. Kumar A. Mehta V. (2020). Distinctive evidence involved in the role of endocannabinoid signalling in Parkinson’s disease: A perspective on associated therapeutic interventions. Int. J. Mol. Sci. 21, E6235. 10.3390/ijms21176235 PMC7504186 32872273

[B15] Bevins R. A. Besheer J. (2006). Object recognition in rats and mice: A one-trial non-matching-to-sample learning task to study 'recognition memory'. Nat. Protoc. 1 (3), 1306–1311. 10.1038/nprot.2006.205 17406415

[B16] Blandini F. (2001). The role of the subthalamic nucleus in the pathophysiology of Parkinson’s disease. Funct. Neurol. 16, 99–106. 11996537

[B17] Bogdanov M. Brown R. H. Matson W. Smart R. Hayden D. O’Donnell H. (2000). Increased oxidative damage to DNA in ALS patients. Free Radic. Biol. Med. 29 (7), 652–658. 10.1016/s0891-5849(00)00349-x 11033417

[B18] Bonda D. J. Wang X. Perry G. Nunomura A. Tabaton M. Zhu X. (2010). Oxidative stress in alzheimer disease: A possibility for prevention. Neuropharmacology 59 (4-5), 290–294. 10.1016/j.neuropharm.2010.04.005 20394761

[B19] Bossy-Wetzel E. Petrilli A. Knott A. B. (2008). Mutant huntingtin and mitochondrial dysfunction. Trends Neurosci. 31 (12), 609–616. 10.1016/j.tins.2008.09.004 18951640 PMC2613540

[B20] Boyina H. K. Jerald M. K. Bharatraj D. K. Diwan P. V. (2018). Influence of fisetin combined with hesperidin on chronic mild hyperhomocysteinemia induced cognitive dysfunction and oxidative stress in Wistar rats. PharmaNutrition 6 (3), 125–136. 10.1016/j.phanu.2018.06.003

[B21] Brichta L. Greengard P. (2014). Molecular determinants of selective dopaminergic vulnerability in Parkinson’s disease: An update. Front. Neuroanat. 8, 152. 10.3389/fnana.2014.00152 25565977 PMC4266033

[B22] Brustovetsky N. Brustovetsky T. Purl K. J. Capano M. Crompton M. Dubinsky J. M. (2003). Increased susceptibility of striatal mitochondria to calcium-induced permeability transition. J. Neurosci. 23 (12), 4858–4867. 10.1523/JNEUROSCI.23-12-04858.2003 12832508 PMC6741171

[B23] Burdo J. Schubert D. Maher P. (2008). Glutathione production is regulated via distinct pathways in stressed and non-stressed cortical neurons. Brain Res. 1189, 12–22. 10.1016/j.brainres.2007.10.077 18048013 PMC2635888

[B24] Cai K. Wang F. Lu J.-Q. Shen A.-N. Zhao S.-M. Zang W.-D. (2022). Nicotinamide mononucleotide alleviates cardiomyopathy phenotypes caused by short-chain enoyl-coa hydratase 1 deficiency. JACC. Basic Transl. Sci. 7, 348–362. 10.1016/j.jacbts.2021.12.007 35540099 PMC9079797

[B25] Carriedo S. G. Sensi S. L. Yin H. Z. Weiss J. H. (2000). AMPA exposures induce mitochondrial Ca 2+ overload and ROS generation in spinal motor neurons *in vitro* . J. Neurosci. 20, 240–250. 10.1523/JNEUROSCI.20-01-00240.2000 10627601 PMC6774118

[B26] Chen C. Kong A. N. (2004). Dietary chemopreventive compounds and ARE/EpRE signaling. Free Radic. Biol. Med. 36 (12), 1505–1516. 10.1016/j.freeradbiomed.2004.03.015 15182853

[B27] Chen C. Yao L. Cui J. Liu B. (2018). Fisetin protects against intracerebral hemorrhage-induced neuroinflammation in aged mice. Cerebrovasc. Dis. 45 (3-4), 154–161. 10.1159/000488117 29587289

[B28] Chen L. Na R. Gu M. Richardson A. Ran Q. (2008). Lipid peroxidation up‐regulates BACE1 expression *in vivo*: A possible early event of amyloidogenesis in Alzheimer’s disease. J. Neurochem. 107 (1), 197–207. 10.1111/j.1471-4159.2008.05603.x 18680556 PMC2716044

[B29] Chen T. J. Feng Y. Liu T. Wu T. T. Chen Y. J. Li X. (2020). Fisetin regulates gut microbiota and exerts neuroprotective effect on mouse model of Parkinson’s disease. Front. Neurosci. 14, 549037. 10.3389/fnins.2020.549037 33381005 PMC7768012

[B30] Chen X. Guo C. Kong J. (2012). Oxidative stress in neurodegenerative diseases. Neural Regen. Res. 7 (5), 376–385. 10.3969/j.issn.1673-5374.2012.05.009 25774178 PMC4350122

[B31] Cho N. Lee K. Y. Huh J. Choi J. H. Yang H. Jeong E. J. (2013). Cognitive-enhancing effects of Rhus verniciflua bark extract and its active flavonoids with neuroprotective and anti-inflammatory activities. Food Chem. Toxicol. 58, 355–361. 10.1016/j.fct.2013.05.007 23688860

[B32] Choubey P. Kwatra M. Pandey S. N. Kumar D. Dwivedi D. K. Rajput P. (2019). Ameliorative effect of fisetin against lipopolysaccharide and restraint stress-induced behavioral deficits via modulation of NF-κB and Ido-1. Psychopharmacology 236 (2), 741–752. 10.1007/s00213-018-5105-3 30426184

[B33] Choudhary S. Kumar P. Malik J. (2013). Plants and phytochemicals for Huntington’s disease. Pharmacogn. Rev. 7 (14), 81–91. 10.4103/0973-7847.120505 24347915 PMC3841999

[B34] Chuang J. Y. Chang P. C. Shen Y. C. Lin C. Tsai C. F. Chen J. H. (2014). Regulatory effects of fisetin on microglial activation. Molecules 19 (7), 8820–8839. 10.3390/molecules19078820 24972270 PMC6271444

[B35] Cordaro M. D’Amico R. Fusco R. Peritore A. F. Genovese T. Interdonato L. (2022). Discovering the effects of fisetin on NF-κB/NLRP-3/NRF-2 molecular pathways in a mouse model of vascular dementia induced by repeated bilateral carotid occlusion. Biomedicines 10. 10.3390/biomedicines10061448 PMC9221103 35740470

[B36] Cos P. Ying L. Calomme M. Hu J. P. Cimanga K. Van Poel B. (1998). Structure–activity relationship and classification of flavonoids as inhibitors of xanthine oxidase and superoxide scavengers. J. Nat. Prod. 61 (1), 71–76. 10.1021/np970237h 9461655

[B37] Covarrubias-Pinto A. Moll P. Solís-Maldonado M. Acuña A. I. Riveros A. Miró M. P. (2015). Beyond the redox imbalance: Oxidative stress contributes to an impaired GLUT3 modulation in Huntington’s disease. Free Radic. Biol. Med. 89, 1085–1096. 10.1016/j.freeradbiomed.2015.09.024 26456058 PMC4840472

[B38] Cox C. Choudhry F. Peacey E. Perkinton M. Richardson J. Howlett D. (2014). Dietary (-) epicatechin as a potent inhibitor of βγ-secretase APP processing. 10.1016/j.neurobiolaging.2014.07.032 PMC4270442 25316600

[B39] Currais A. Farrokhi C. Dargusch R. Armando A. Quehenberger O. Schubert D. (2018). Fisetin reduces the impact of aging on behavior and physiology in the rapidly aging SAMP8 mouse. J. Gerontol. A Biol. Sci. Med. Sci. 73 (3), 299–307. 10.1093/gerona/glx104 28575152 PMC5861950

[B40] Currais A. Maher P. (2013). Functional consequences of age-dependent changes in glutathione status in the brain. Antioxid. Redox Signal. 19 (8), 813–822. 10.1089/ars.2012.4996 23249101

[B41] Currais A. Prior M. Dargusch R. Armando A. Ehren J. Schubert D. (2014). Modulation of p25 and inflammatory pathways by fisetin maintains cognitive function in A lzheimer’s disease transgenic mice. Aging Cell. 13 (2), 379–390. 10.1111/acel.12185 24341874 PMC3954948

[B42] Dalfó E. Portero-Otín M. Ayala V. Martínez A. Pamplona R. Ferrer I. (2005). Evidence of oxidative stress in the neocortex in incidental Lewy Body disease. J. Neuropathol. Exp. Neurol. 64 (9), 816–830. 10.1097/01.jnen.0000179050.54522.5a 16141792

[B43] D’Amico E. Factor-Litvak P. Santella R. M. Mitsumoto H. (2013). Clinical perspective on oxidative stress in sporadic amyotrophic lateral sclerosis. Free Radic. Biol. Med. 65, 509–527. 10.1016/j.freeradbiomed.2013.06.029 23797033 PMC3859834

[B44] Dash R. Emran T. B. Uddin M. M. Islam A. Junaid M. (2014). Molecular docking of fisetin with AD associated AChE, ABAD and BACE1 proteins. Bioinformation 10 (9), 562–568. 10.6026/97320630010562 25352723 PMC4209364

[B45] Dávila D. Torres-Aleman I. (2008). Neuronal death by oxidative stress involves activation of FOXO3 through a two-arm pathway that activates stress kinases and attenuates insulin-like growth factor I signaling. Mol. Biol. Cell. 19 (5), 2014–2025. 10.1091/mbc.e07-08-0811 18287535 PMC2366852

[B46] de Abreu Costa M. D’Alò de Oliveira G. S. D. A. Tatton-Ramos T. Manfro G. G. Salum G. A. (2019). Anxiety and stress-related disorders and mindfulness-based interventions: A systematic review and multilevel meta-analysis and meta-regression of multiple outcomes. Mindfulness 10 (6), 996–1005. 10.1007/s12671-018-1058-1

[B47] de Vries H. E. Witte M. Hondius D. Rozemuller A. J. Drukarch B. Hoozemans J. (2008). Nrf2-induced antioxidant protection: A promising target to counteract ROS-mediated damage in neurodegenerative disease? Free Radic. Biol. Med. 45 (10), 1375–1383. 10.1016/j.freeradbiomed.2008.09.001 18824091

[B48] Deister C. A. Teagarden M. A. WilsonPaladini C. J. C. A. (2009). An intrinsic neuronal oscillator underlies dopaminergic neuron bursting. J. Neurosci. 29, 15888–15897. 10.1523/JNEUROSCI.4053-09.2009 20016105 PMC2824818

[B49] Dias V. Junn E. Mouradian M. M. (2013). The role of oxidative stress in Parkinson’s disease. J. Park. Dis. 3 (4), 461–491. 10.3233/JPD-130230 PMC4135313 24252804

[B50] Dimitrić Marković J. M. D. Marković Z. S. Brdarić T. P. Filipović N. D. (2011). Comparative spectroscopic and mechanistic study of chelation properties of fisetin with iron in aqueous buffered solutions. Implications on *in vitro* antioxidant activity. Dalton Trans. 40 (17), 4560–4571. 10.1039/c0dt01834a 21431152

[B51] Duan C. Deng H. Xiao S. Xie J. Li H. Zhao X. (2022). Accelerate gas diffusion-weighted MRI for lung morphometry with deep learning. Eur. Radiol. 32, 702–713. 10.1007/s00330-021-08126-y 34255160 PMC8276538

[B52] Ehren J. L. Maher P. (2013). Concurrent regulation of the transcription factors Nrf2 and ATF4 mediates the enhancement of glutathione levels by the flavonoid fisetin. Biochem. Pharmacol. 85, 1816–1826. 10.1016/j.bcp.2013.04.010 23618921

[B53] Ehrlich M. E. (2012). Huntington’s disease and the striatal medium spiny neuron: Cell-autonomous and non-cell-autonomous mechanisms of disease. Neurotherapeutics 9 (2), 270–284. 10.1007/s13311-012-0112-2 22441874 PMC3337013

[B54] Estrada Sánchez A. M. Mejía-Toiber J. Massieu L. (2008). Excitotoxic neuronal death and the pathogenesis of Huntington’s disease. Arch. Med. Res. 39 (3), 265–276. 10.1016/j.arcmed.2007.11.011 18279698

[B55] Ferber S. Namdar D. Hen-Shoval D. Eger G. Koltai H. Shoval G. (2019). The entourage effect: Terpenes coupled with cannabinoids for the treatment of mood disorders and anxiety disorders. Curr. Neuropharmacol. 18, 87–96. 10.2174/1570159X17666190903103923 PMC7324885 31481004

[B56] Ferrari F. Villa R. F. (2017). The neurobiology of depression: An integrated overview from biological theories to clinical evidence. Mol. Neurobiol. 54 (7), 4847–4865. 10.1007/s12035-016-0032-y 27510505

[B57] Ferreira-Vieira T. H. Guimaraes I. M. Silva F. R. Ribeiro F. M. (2016). Alzheimer’s disease: Targeting the cholinergic system. Curr. Neuropharmacol. 14 (1), 101–115. 10.2174/1570159x13666150716165726 26813123 PMC4787279

[B58] Fischer M. T. Wimmer I. Höftberger R. Gerlach S. Haider L. Zrzavy T. (2013). Disease-specific molecular events in cortical multiple sclerosis lesions. Brain 136 (6), 1799–1815. 10.1093/brain/awt110 23687122 PMC3673462

[B59] Fischer W. Currais A. Liang Z. Pinto A. Maher P. (2019). Old age-associated phenotypic screening for Alzheimer’s disease drug candidates identifies sterubin as a potent neuroprotective compound from Yerba Santa. Redox Biol. 21, 101089. 10.1016/j.redox.2018.101089 30594901 PMC6309122

[B60] Förstl H. Kurz A. (1999). Clinical features of Alzheimer’s disease. Eur. Arch. Psychiatry Clin. Neurosci. 249, 288–290. 10.1007/s004060050101 10653284

[B61] Gamez J. Corbera-Bellalta M. Nogales G. Raguer N. García-Arumí E. Badia-Canto M. (2006). Mutational analysis of the Cu/Zn superoxide dismutase gene in a Catalan ALS population: Should all sporadic ALS cases also be screened for SOD1? J. Neurol. Sci. 247 (1), 21–28. 10.1016/j.jns.2006.03.006 16674979

[B62] Gangemi S. Gofita E. Costa C. Teodoro M. Briguglio G. Nikitovic D. (2016). Occupational and environmental exposure to pesticides and cytokine pathways in chronic diseases (Review). Int. J. Mol. Med. 38 (4), 1012–1020. 10.3892/ijmm.2016.2728 27600395 PMC5029960

[B63] Ganguly G. Chakrabarti S. Chatterjee U. Saso L. (2017). Proteinopathy, oxidative stress and mitochondrial dysfunction: Cross talk in Alzheimer’s disease and Parkinson’s disease. Drug Des. devel. Ther. 11, 797–810. 10.2147/DDDT.S130514 PMC5358994 28352155

[B64] Gao H. M. Liu B. Hong J. S. (2003). Critical role for microglial NADPH oxidase in rotenone-induced degeneration of dopaminergic neurons. J. Neurosci. 23 (15), 6181–6187. 10.1523/jneurosci.23-15-06181.2003 12867501 PMC6740554

[B65] Giamogante F. Marrocco I. Romaniello D. Eufemi M. Chichiarelli S. Altieri F. (2016). Comparative analysis of the interaction between different flavonoids and PDIA3. Oxid. Med. Cell. Longev. 2016, 4518281. 10.1155/2016/4518281 28044092 PMC5164911

[B66] Gilgun-Sherki Y. Melamed E. Offen D. (2001). Oxidative stress induced-neurodegenerative diseases: The need for antioxidants that penetrate the blood brain barrier. Neuropharmacology 40 (8), 959–975. 10.1016/s0028-3908(01)00019-3 11406187

[B67] Giraldo E. Lloret A. Fuchsberger T. Viña J. (2014). Aβ and tau toxicities in Alzheimer’s are linked via oxidative stress-induced p38 activation: Protective role of vitamin E. Redox Biol. 2, 873–877. 10.1016/j.redox.2014.03.002 25061569 PMC4099506

[B68] Gitler A. D. Dhillon P. Shorter J. (2017). Neurodegenerative disease: Models, mechanisms, and a new hope. Dis. Model. Mech. 10 (5), 499–502. 10.1242/dmm.030205 28468935 PMC5451177

[B69] Goedert M. Spillantini M. G. (2006). A century of Alzheimer’s disease. Science 314 (5800), 777–781. 10.1126/science.1132814 17082447

[B70] Grynkiewicz G. Demchuk O. M. (2019). New perspectives for fisetin. Front. Chem. 7, 697. 10.3389/fchem.2019.00697 31750288 PMC6842927

[B71] Gu M. Gash M. T. Mann V. M. Javoy-Agid F. Cooper J. M. Schapira A. H. V. (1996). Mitochondrial defect in Huntington’s disease caudate nucleus. Ann. Neurol. 39, 385–389. 10.1002/ana.410390317 8602759

[B72] Guan J. Z. Guan W. P. Maeda T. Guoqing X. GuangZhi W. Makino N. (2015). Patients with multiple sclerosis show increased oxidative stress markers and somatic telomere length shortening. Mol. Cell. Biochem. 400 (1-2), 183–187. 10.1007/s11010-014-2274-1 25424527

[B73] Guo P. Feng Y-Y. (2017). Anti-inflammatory effects of kaempferol, myricetin, fisetin and ibuprofen in neonatal rats. Trop. J. Pharm. Res. 16 (8), 1819–1826. 10.4314/tjpr.v16i8.10

[B74] Gupta S. C. Prasad S. Aggarwal B. B. (2016). Anti-inflammatory nutraceuticals and chronic diseases, 928. Springer.

[B75] Guzzo M. R. Uemi M. Donate P. M. Nikolaou S. Machado A. E. Okano L. T. (2006). Study of the complexation of fisetin with cyclodextrins. J. Phys. Chem. A 110 (36), 10545–10551. 10.1021/jp0613337 16956235

[B76] Haddad D. Nakamura K. (2015). Understanding the susceptibility of dopamine neurons to mitochondrial stressors in Parkinson’s disease. FEBS Lett. 589, 3702–3713. 10.1016/j.febslet.2015.10.021 26526613 PMC4679488

[B77] Haider L. Fischer M. T. Frischer J. M. Bauer J. Höftberger R. Botond G. (2011). Oxidative damage in multiple sclerosis lesions. Brain 134 (7), 1914–1924. 10.1093/brain/awr128 21653539 PMC3122372

[B78] Hao P. Li H. Zhou L. Sun H. Han J. Zhang Z. (2022). Serum metal ion-induced cross-linking of photoelectrochemical peptides and circulating proteins for evaluating cardiac ischemia/reperfusion. ACS Sens. 7, 775–783. 10.1021/acssensors.1c02305 35293731

[B79] He W. B. Abe K. Akaishi T. (2018). Oral administration of fisetin promotes the induction of hippocampal long-term potentiation *in vivo* . J. Pharmacol. Sci. 136 (1), 42–45. 10.1016/j.jphs.2017.12.008 29317180

[B80] Hemanth Kumar B. Arun Reddy R. Mahesh Kumar J. Dinesh Kumar B. Diwan P. V. (2017). Effects of fisetin on hyperhomocysteinemia-induced experimental endothelial dysfunction and vascular dementia. Can. J. Physiol. Pharmacol. 95 (1), 32–42. 10.1139/cjpp-2016-0147 27901381

[B81] Hendriks J. J. A. de Vries H. E. van der Pol S. M. A. van den Berg T. K. van Tol E. A. F. Dijkstra C. D. (2003). Flavonoids inhibit myelin phagocytosis by macrophages; a structure–activity relationship study. Biochem. Pharmacol. 65 (5), 877–885. 10.1016/s0006-2952(02)01609-x 12628496

[B82] Hori Y. Takeda S. Cho H. Wegmann S. Shoup T. M. Takahashi K. (2015). A Food and Drug Administration-approved asthma therapeutic agent impacts amyloid β in the brain in a transgenic model of Alzheimer disease. J. Biol. Chem. 290 (4), 1966–1978. 10.1074/jbc.M114.586602 25468905 PMC4303653

[B83] Horwitz R. J. Chapter 30 (2018). “The allergic patient,” in Integrative medicine. Editor Rakel D. 4th ed. (Elsevier), 300–309.e302.

[B84] Hou D. X. Fukuda M. Johnson J. A. Miyamori K. Ushikai M. Fujii M. (2001). Fisetin induces transcription of NADPH: Quinone oxidoreductase gene through an antioxidant responsive element-involved activation. Int. J. Oncol. 18 (6), 1175–1179. 10.3892/ijo.18.6.1175 11351248

[B85] Hritcu L. Ionita R. Postu P. A. Gupta G. K. Turkez H. Lima T. C. (2017). Antidepressant flavonoids and their relationship with oxidative stress. Oxid. Med. Cell. Longev. 2017, 5762172. 10.1155/2017/5762172 29410733 PMC5749298

[B86] Huang M. C. Hsueh T. Y. Cheng Y. Y. Lin L. C. Tsai T. H. (2018). Pharmacokinetics and biliary excretion of fisetin in rats. J. Agric. Food Chem. 66 (25), 6300–6307. 10.1021/acs.jafc.8b00917 29862816

[B87] Hussain T. Al-Attas O. S. Alamery S. Ahmed M. Odeibat H. A. M. Alrokayan S. (2019). The plant flavonoid, fisetin alleviates cigarette smoke‐induced oxidative stress, and inflammation in Wistar rat lungs. J. Food Biochem. 43 (8), e12962. 10.1111/jfbc.12962 31368542

[B88] Ishige K. Schubert D. Sagara Y. (2001). Flavonoids protect neuronal cells from oxidative stress by three distinct mechanisms. Free Radic. Biol. Med. 30 (4), 433–446. 10.1016/s0891-5849(00)00498-6 11182299

[B89] Ishizawa T. Mattila P. Davies P. Wang D. Dickson D. W. (2003). Colocalization of tau and alpha-synuclein epitopes in Lewy bodies. J. Neuropathol. Exp. Neurol. 62 (4), 389–397. 10.1093/jnen/62.4.389 12722831

[B90] Jacob S. Thangarajan S. (2017). Effect of gestational intake of fisetin (3, 3′, 4′, 7-tetrahydroxyflavone) on developmental methyl mercury neurotoxicity in F 1 generation rats. Biol. Trace Elem. Res. 177 (2), 297–315. 10.1007/s12011-016-0886-x 27815688

[B91] Jash S. K. Mondal S. (2014). Bioactive flavonoid fisetin–A molecule of pharmacological interest. Cardiovasc Dis. 5, 6.

[B92] Jeong E. J. Ma C. J. Lee K. Y. Kim S. H. Sung S. H. Kim Y. C. (2009). KD-501, a standardized extract of Scrophularia buergeriana has both cognitive-enhancing and antioxidant activities in mice given scopolamine. J. Ethnopharmacol. 121 (1), 98–105. 10.1016/j.jep.2008.10.006 18996178

[B93] Jhonsa D. Badgujar L. B. Sutariya B. Saraf M. N. (2016). Neuroprotective effect of flavonoids against paraquat induced oxidative stress and neurotoxicity in *Drosophila melanogaster* . Curr. Top. Nutraceutical Res. 14 (4).

[B94] Jin K. Yan Y. Chen M. Wang J. Pan X. Liu X. (2022). Multimodal deep learning with feature level fusion for identification of choroidal neovascularization activity in age-related macular degeneration. Acta Ophthalmol. 100, e512e512–e520. 10.1111/aos.14928 34159761

[B95] Jo J. H. Jo J. J. Lee J. M. Lee S. (2016). Identification of absolute conversion to geraldol from fisetin and pharmacokinetics in mouse. J. Chromatogr. B Anal. Technol. Biomed. Life Sci. 1038, 95–100. 10.1016/j.jchromb.2016.10.034 27810278

[B96] Kadari A. Gudem S. Kulhari H. Bhandi M. M. Borkar R. M. Kolapalli V. R. (2017). Enhanced oral bioavailability and anticancer efficacy of fisetin by encapsulating as inclusion complex with HPβCD in polymeric nanoparticles. Drug Deliv. 24 (1), 224–232. 10.1080/10717544.2016.1245366 28156161 PMC8241160

[B97] Kalia L. V. Lang A. E. (2015). Parkinson’s disease. Lancet 386 (9996), 896–912. 10.1016/S0140-6736(14)61393-3 25904081

[B98] Kang K. A. Piao M. J. Kim K. C. Cha J. W. Zheng J. Yao C. W. (2014). Fisetin attenuates hydrogen peroxide-induced cell damage by scavenging reactive oxygen species and activating protective functions of cellular glutathione system. Vitro Cell. Dev. Biol. Anim. 50 (1), 66–74. 10.1007/s11626-013-9681-6 23982916

[B99] Karthika C. Appu A. P. Akter R. Rahman M. H. Tagde P. Ashraf G. M. (2022). Potential innovation against Alzheimer’s disorder: A tricomponent combination of natural antioxidants (vitamin E, quercetin, and basil oil) and the development of its intranasal delivery. Environ. Sci. Pollut. Res. Int. 29, 10950–10965. 10.1007/s11356-021-17830-7 35000160

[B100] Kasprzak M. M. Erxleben A. Ochocki J. (2015). Properties and applications of flavonoid metal complexes. RSC Adv. 5 (57), 45853–45877. 10.1039/C5RA05069C

[B101] Katalini M. Bosak A. Kovarik Z. (2014). Flavonoids as inhibitors of human butyrylcholinesterase variants. Food Technol. Biotechnol. 52 (1), 64.

[B102] Kaur D. Behl T. Sehgal A. Singh S. Sharma N. Badavath V. N. (2022). Unravelling the potential neuroprotective facets of erythropoietin for the treatment of Alzheimer’s disease. Metab. Brain Dis. 37, 1–16. 10.1007/s11011-021-00820-6 34436747

[B103] Kesarwani P. Murali A. K. Al-Khami A. A. Mehrotra S. (2013). Redox regulation of T-cell function: From molecular mechanisms to significance in human health and disease. Antioxid. Redox Signal. 18 (12), 1497–1534. 10.1089/ars.2011.4073 22938635 PMC3603502

[B104] Khan N. Syed D. N. Ahmad N. Mukhtar H. (2013). Fisetin: A dietary antioxidant for health promotion. Antioxid. Redox Signal. 19 (2), 151–162. 10.1089/ars.2012.4901 23121441 PMC3689181

[B105] Kim J. H. Kim M. Y. Kim J. H. Cho J. Y. (2015). Fisetin suppresses macrophage-mediated inflammatory responses by blockade of Src and Syk. Biomol. Ther. 23 (5), 414–420. 10.4062/biomolther.2015.036 PMC4556200 26336580

[B106] Kim S. Choi K. J. Cho S. J. Yun S. M. Jeon J. P. Koh Y. H. (2016). Fisetin stimulates autophagic degradation of phosphorylated tau via the activation of TFEB and Nrf2 transcription factors. Sci. Rep. 6 (1), 24933. 10.1038/srep24933 27112200 PMC4844953

[B107] Kin K. Yasuhara T. Kameda M. Date I. (2019). Animal models for Parkinson’s disease research: Trends in the 2000s. Int. J. Mol. Sci. 20 (21), E5402. 10.3390/ijms20215402 PMC6862023 31671557

[B108] Krasieva T. B. Ehren J. O’Sullivan T. Tromberg B. J. Maher P. (2015). Cell and brain tissue imaging of the flavonoid fisetin using label-free two-photon microscopy. Neurochem. Int. 89, 243–248. 10.1016/j.neuint.2015.08.003 26271433 PMC4587296

[B109] Kriete A. Mayo K. L. (2009). Atypical pathways of NF-kappaB activation and aging. Exp. Gerontol. 44 (4), 250–255. 10.1016/j.exger.2008.12.005 19174186

[B110] Kumar A. Singh A. Ekavali (2015). A review on Alzheimer’s disease pathophysiology and its management: An update. Pharmacol. Rep. 67 (2), 195–203. 10.1016/j.pharep.2014.09.004 25712639

[B111] Kumar B. H. Reddy R. A. Kumar J. M. Kumar B. D. Diwan P. V. (2017). Effects of fisetin on hyperhomocysteinemia-induced experimental endothelial dysfunction and vascular dementia. Can. J. Physiol. Pharmacol. 95, 32–42. 10.1139/cjpp-2016-0147 27901381

[B112] Kumar R. Kumar R. Khurana N. Singh S. K. Khurana S. Verma S. (2020). Enhanced oral bioavailability and neuroprotective effect of fisetin through its SNEDDS against rotenone-induced Parkinson’s disease rat model. Food Chem. Toxicol. 144, 111590. 10.1016/j.fct.2020.111590 32710995

[B113] Kure S. Tominaga T. Yoshimoto T. Tada K. Narisawa K. (1991). Glutamate triggers internucleosomal DNA cleavage in neuronal cells. Biochem. Biophys. Res. Commun. 179, 39–45. 10.1016/0006-291X(91)91330-F 1679329

[B114] Lee S. E. Jeong S. I. Yang H. Park C. S. Jin Y. H. Park Y. S. (2011). Fisetin induces Nrf2-mediated HO-1 expression through PKC-δ and p38 in human umbilical vein endothelial cells. J. Cell. Biochem. 112 (9), 2352–2360. 10.1002/jcb.23158 21520244

[B115] Léotoing L. Wauquier F. Guicheux J. Miot-Noirault E. Wittrant Y. Coxam V. (2013). The polyphenol fisetin protects bone by repressing NF-κB and MKP-1-dependent signaling pathways in osteoclasts. PLOS ONE 8 (7), e68388. 10.1371/journal.pone.0068388 23861901 PMC3701685

[B116] Li H. Zhao X. Wang Y. Lou X. Chen S. Deng H. (2021). Damaged lung gas exchange function of discharged COVID-19 patients detected by hyperpolarized (129)Xe MRI. Sci. Adv. 7, eabc8180. 10.1126/sciadv.abc8180 33219111 PMC7775756

[B117] Li Q. Spencer N. Y. Pantazis N. J. Engelhardt J. F. (2011). Alsin and SOD1G93A proteins regulate endosomal reactive oxygen species production by glial cells and proinflammatory pathways responsible for neurotoxicity. J. Biol. Chem. 286 (46), 40151–40162. 10.1074/jbc.M111.279711 21937428 PMC3220533

[B118] Li Z. Teng M. Yang R. Lin F. Fu Y. Lin W. (2022). Sb-doped WO3 based QCM humidity sensor with self-recovery ability for real-time monitoring of respiration and wound. Sensors Actuators B Chem. 361, 131691. 10.1016/j.snb.2022.131691

[B119] Liebsch F. Kulic L. Teunissen C. Shobo A. Ulku I. Engelschalt V. (2019). Aβ34 is a BACE1-derived degradation intermediate associated with amyloid clearance and Alzheimer’s disease progression. Nat. Commun. 10 (1), 2240. 10.1038/s41467-019-10152-w 31110178 PMC6527709

[B120] Lin M. T. Beal M. F. (2006). Mitochondrial dysfunction and oxidative stress in neurodegenerative diseases. Nature 443 (7113), 787–795. 10.1038/nature05292 17051205

[B121] Lin M. T. Lin C. L. Lin T. Y. Cheng C. W. Yang S. F. Lin C. L. (2016). Synergistic effect of fisetin combined with sorafenib in human cervical cancer HeLa cells through activation of death receptor-5 mediated caspase-8/caspase-3 and the mitochondria-dependent apoptotic pathway. Tumour Biol. 37 (5), 6987–6996. 10.1007/s13277-015-4526-4 26662956

[B122] Liot G. Valette J. Pépin J. Flament J. Brouillet E. (2017). Energy defects in Huntington’s disease: Why ”*in vivo*” evidence matters. Biochem. Biophys. Res. Commun. 483 (4), 1084–1095. 10.1016/j.bbrc.2016.09.065 27639641

[B123] Liu C. Wang Y. Li L. He D. Chi J. Li Q. (2022). Engineered extracellular vesicles and their mimetics for cancer immunotherapy. J. Control. Release 349, 679–698. 10.1016/j.jconrel.2022.05.062 35878728

[B124] Liu Z. Su W. Ao J. Wang M. Jiang Q. He J. (2022b). Instant diagnosis of gastroscopic biopsy via deep-learned single-shot femtosecond stimulated Raman histology. Nat. Commun. 13, 4050. 10.1038/s41467-022-31339-8 35831299 PMC9279377

[B125] Llorens-Martín M. Jurado J. Hernández F. Avila J. (2014). GSK-3β, a pivotal kinase in Alzheimer disease. Front. Mol. Neurosci. 7, 46. 10.3389/fnmol.2014.00046 24904272 PMC4033045

[B126] Luthi-Carter R. Strand A. Peters N. L. Solano S. M. Hollingsworth Z. R. Menon A. S. (2000). Decreased expression of striatal signaling genes in a mouse model of Huntington’s disease. Hum. Mol. Genet. 9 (9), 1259–1271. 10.1093/hmg/9.9.1259 10814708

[B127] Magesh S. Chen Y. Hu L. (2012). Small molecule modulators of Keap1-Nrf2-ARE pathway as potential preventive and therapeutic agents. Med. Res. Rev. 32 (4), 687–726. 10.1002/med.21257 22549716 PMC3393814

[B128] Maher P. Akaishi T. Abe K. (2006). Flavonoid fisetin promotes ERK-dependent long-term potentiation and enhances memory. Proc. Natl. Acad. Sci. U. S. A. 103 (44), 16568–16573. 10.1073/pnas.0607822103 17050681 PMC1637622

[B129] Maher P. Dargusch R. Bodai L. Gerard P. E. Purcell J. M. Marsh J. L. (2011a). ERK activation by the polyphenols fisetin and resveratrol provides neuroprotection in multiple models of Huntington’s disease. Hum. Mol. Genet. 20 (2), 261–270. 10.1093/hmg/ddq460 20952447 PMC3005900

[B130] Maher P. Dargusch R. Ehren J. L. Okada S. Sharma K. Schubert D. (2011b). Fisetin lowers methylglyoxal dependent protein glycation and limits the complications of diabetes. PLOS ONE 6 (6), e21226. 10.1371/journal.pone.0021226 21738623 PMC3124487

[B131] Maher P. Hanneken A. (2005). Flavonoids protect retinal ganglion cells from oxidative stress–induced death. Investig. Ophthalmol. Vis. Sci. 46 (12), 4796–4803. 10.1167/iovs.05-0397 16303981

[B132] Maher P. Kontoghiorghes G. J. (2015). Characterization of the neuroprotective potential of derivatives of the iron chelating drug deferiprone. Neurochem. Res. 40 (3), 609–620. 10.1007/s11064-014-1508-7 25559767

[B133] Maher P. (2006). A comparison of the neurotrophic activities of the flavonoid fisetin and some of its derivatives. Free Radic. Res. 40 (10), 1105–1111. 10.1080/10715760600672509 17015255

[B134] Maher P. (2015). How fisetin reduces the impact of age and disease on CNS function. Front. Biosci. 7, 58–82. 10.2741/S425 PMC5527824 25961687

[B135] Maher P. (2009). Modulation of multiple pathways involved in the maintenance of neuronal function during aging by fisetin. Genes. Nutr. 4 (4), 297–307. 10.1007/s12263-009-0142-5 19756810 PMC2775892

[B136] Maher P. (2020). Preventing and treating neurological disorders with the flavonol fisetin. Brain Plast. 6 (2), 155–166. 10.3233/BPL-200104 PMC7990461 33782648

[B137] Maher P. (2017). Protective effects of fisetin and other berry flavonoids in Parkinson’s disease. Food Funct. 8 (9), 3033–3042. 10.1039/c7fo00809k 28714503

[B138] Maher P. (2008). The flavonoid fisetin promotes nerve cell survival from trophic factor withdrawal by enhancement of proteasome activity. Arch. Biochem. Biophys. 476 (2), 139–144. 10.1016/j.abb.2008.03.023 18396148

[B139] Maher P. (2019). The potential of flavonoids for the treatment of neurodegenerative diseases. Int. J. Mol. Sci. 20 (12), 3056. 10.3390/ijms20123056 31234550 PMC6627573

[B140] Mandemakers W. Morais V. A. De Strooper B. (2007). A cell biological perspective on mitochondrial dysfunction in Parkinson disease and other neurodegenerative diseases. J. Cell. Sci. 120 (10), 1707–1716. 10.1242/jcs.03443 17502481

[B141] Mansuri M. L. Parihar P. Solanki I. Parihar M. S. (2014). Flavonoids in modulation of cell survival signalling pathways. Genes. Nutr. 9 (3), 400. 10.1007/s12263-014-0400-z 24682883 PMC4026439

[B142] Mattson M. P. Magnus T. (2006). Ageing and neuronal vulnerability. Nat. Rev. Neurosci. 7, 278–294. 10.1038/nrn1886 16552414 PMC3710114

[B143] McLennan H. R. Esposti M. D. (2000). The contribution of mitochondrial respiratory complexes to the production of reactive oxygen species. J. Bioenerg. Biomembr. 32 (2), 153–162. 10.1023/a:1005507913372 11768748

[B144] Meiser J. Weindl D. Hiller K. (2013). Complexity of dopamine metabolism. Cell. Commun. Signal. 11 (1), 34. 10.1186/1478-811X-11-34 23683503 PMC3693914

[B145] Meister A. Anderson M. E. (1983). Annu. Rev. Biochem. 52 (1), 711–760. 10.1146/annurev.bi.52.070183.003431 6137189

[B146] Menzies F. M. Ince P. G. Shaw P. J. (2002). Mitochondrial involvement in amyotrophic lateral sclerosis. Neurochem. Int. 40, 543–551. 10.1016/s0197-0186(01)00125-5 11850111

[B147] Miao J. Shi R. Li L. Chen F. Zhou Y. Tung Y. C. (2019). Pathological tau from Alzheimer’s brain induces site-specific hyperphosphorylation and SDS-and reducing agent-resistant aggregation of tau *in vivo* . Front. Aging Neurosci. 11, 34. 10.3389/fnagi.2019.00034 30890929 PMC6411797

[B148] Michel P. P. Hirsch E. C. Hunot S. (2016). Understanding dopaminergic cell death pathways in Parkinson disease. Neuron 90 (4), 675–691. 10.1016/j.neuron.2016.03.038 27196972

[B149] Mitomi Y. Nomura T. Kurosawa M. Nukina N. Furukawa Y. (2012). Post-aggregation oxidation of mutant huntingtin controls the interactions between aggregates. J. Biol. Chem. 287 (41), 34764–34775. 10.1074/jbc.M112.387035 22891249 PMC3464579

[B150] Momtaz S. Memariani Z. El-Senduny F. F. Sanadgol N. Golab F. Katebi M. (2020). Targeting ubiquitin-proteasome pathway by natural products: Novel therapeutic strategy for treatment of neurodegenerative diseases. Front. Physiol. 11, 361. 10.3389/fphys.2020.00361 32411012 PMC7199656

[B151] Muddapu V. R. Mandali A. Chakravarthy V. S. Ramaswamy S. (2019). A computational model of loss of dopaminergic cells in Parkinson’s disease due to glutamate-induced excitotoxicity. Front. Neural Circuits 13, 11. 10.3389/FNCIR.2019.00011 30858799 PMC6397878

[B152] Mufson E. J. Counts S. E. Perez S. E. Ginsberg S. D. (2008). Cholinergic system during the progression of Alzheimer’s disease: Therapeutic implications. Expert Rev. Neurother. 8 (11), 1703–1718. 10.1586/14737175.8.11.1703 18986241 PMC2631573

[B153] Myara I. Pico I. Vedie B. Moatti N. (1993). A method to screen for the antioxidant effect of compounds on low-density lipoprotein (LDL): Illustration with flavonoids. J. Pharmacol. Toxicol. Methods 30 (2), 69–73. 10.1016/1056-8719(93)90009-4 8298183

[B154] Myhrstad M. C. Carlsen H. Nordström O. Blomhoff R. Jø Moskaug (2002). Flavonoids increase the intracellular glutathione level by transactivation of the γ-glutamylcysteine synthetase catalytical subunit promoter. Free Radic. Biol. Med. 32 (5), 386–393. 10.1016/s0891-5849(01)00812-7 11864778

[B155] Naeimi A. F. Alizadeh M. (2017). Antioxidant properties of the flavonoid fisetin: An updated review of *in vivo* and *in vitro* studies. Trends Food Sci. Technol. 70, 34–44. 10.1016/j.tifs.2017.10.003

[B156] Nakamura T. Lipton S. A. (2010). Preventing Ca2+-mediated nitrosative stress in neurodegenerative diseases: Possible pharmacological strategies. Cell. Calcium 47 (2), 190–197. 10.1016/j.ceca.2009.12.009 20060165 PMC2875138

[B157] Nakamura T. Lipton S. A. (2011). Redox modulation by S-nitrosylation contributes to protein misfolding, mitochondrial dynamics, and neuronal synaptic damage in neurodegenerative diseases. Cell. Death Differ. 18 (9), 1478–1486. 10.1038/cdd.2011.65 21597461 PMC3178424

[B158] Naoi M. Shamoto-Nagai M. Maruyama W. (2019). Neuroprotection of multifunctional phytochemicals as novel therapeutic strategy for neurodegenerative disorders: Antiapoptotic and antiamyloidogenic activities by modulation of cellular signal pathways. Future Neurol. 14 (1), FNL9. 10.2217/fnl-2018-0028

[B159] Nguyen T. Sherratt P. J. Pickett C. B. (2003). Regulatory mechanisms controlling gene expression mediated by the antioxidant response element. Annu. Rev. Pharmacol. Toxicol. 43 (1), 233–260. 10.1146/annurev.pharmtox.43.100901.140229 12359864

[B160] Nizzari M. Thellung S. Corsaro A. Villa V. Pagano A. Porcile C. (2012). Neurodegeneration in alzheimer disease: Role of amyloid precursor protein and presenilin 1 intracellular signaling. J. Toxicol. 2012, 187297. 10.1155/2012/187297 22496686 PMC3306972

[B161] Ohl K. Tenbrock K. Kipp M. (2016). Oxidative stress in multiple sclerosis: Central and peripheral mode of action. Exp. Neurol. 277, 58–67. 10.1016/j.expneurol.2015.11.010 26626971 PMC7094520

[B162] Orhan I. E. Daglia M. Nabavi S. F. Loizzo M. R. Sobarzo-Sánchez E. Nabavi S. M. (2015). Flavonoids and dementia: An update. Curr. Med. Chem. 22 (8), 1004–1015. 10.2174/0929867322666141212122352 25515512

[B163] Oteiza P. I. Uchitel O. D. Carrasquedo F. Dubrovski A. L. Roma J. C. Fraga C. G. (1997). Evaluation of antioxidants, protein, and lipid oxidation products in blood from sporadic amyotrophic lateral sclerosis patients. Neurochem. Res. 22 (4), 535–539. 10.1023/a:1027384432715 9130267

[B164] Ott M. Gogvadze V. Orrenius S. Zhivotovsky B. (2007). Mitochondria, oxidative stress and cell death. Apoptosis 12 (5), 913–922. 10.1007/s10495-007-0756-2 17453160

[B165] Pacelli C. Giguère N. Bourque M. J. Lévesque M. Slack R. S. Trudeau L. É. (2015). Elevated mitochondrial bioenergetics and axonal arborization size are key contributors to the vulnerability of dopamine neurons. Curr. Biol. 25 (18), 2349–2360. 10.1016/j.cub.2015.07.050 26320949

[B166] Pal H. C. Pearlman R. L. Afaq F. (2016). Fisetin and its role in chronic diseases. Adv. Exp. Med. Biol. 928, 213–244. 10.1007/978-3-319-41334-1_10 27671819

[B167] Patel M. Y. Panchal H. V. Ghribi O. Benzeroual K. E. (2012). The neuroprotective effect of fisetin in the MPTP model of Parkinson’s disease. J. Park. Dis. 2 (4), 287–302. 10.3233/JPD-012110 23938259

[B168] Patten D. A. Germain M. Kelly M. A. Slack R. S. (2010). Reactive oxygen species: Stuck in the middle of neurodegeneration. J. Alzheimers Dis. 20 (2), S357–S367. 10.3233/JAD-2010-100498 20421690

[B169] Piao M. J. Kim K. C. Chae S. Keum Y. S. Kim H. S. Hyun J. W. (2013). Protective effect of fisetin (3, 7, 3′, 4′-tetrahydroxyflavone) against γ-irradiation-induced oxidative stress and cell damage. Biomol. Ther. 21 (3), 210–215. 10.4062/biomolther.2013.017 PMC3830119 24265866

[B170] Pitts A. Dailey K. Newington J. T. Chien A. Arseneault R. Cann T. (2012). Dithiol-based compounds maintain expression of antioxidant protein peroxiredoxin 1 that counteracts toxicity of mutant huntingtin. J. Biol. Chem. 287 (27), 22717–22729. 10.1074/jbc.M111.334565 22577145 PMC3391089

[B171] Pizzino G. Irrera N. Cucinotta M. Pallio G. Mannino F. Arcoraci V. (2017). Oxidative stress: Harms and benefits for human health. Oxid. Med. Cell. Longev. 2017, 8416763. 10.1155/2017/8416763 28819546 PMC5551541

[B172] Polymeropoulos M. H. Lavedan C. Leroy E. Ide S. E. Dehejia A. Dutra A. (1997). Mutation in the alpha-synuclein gene identified in families with Parkinson's disease. Science 276 (5321), 2045–2047. 10.1126/science.276.5321.2045 9197268

[B173] Prabhu K. Bhute A. S. (2012). Plant based natural dyes and mordants: A review. J. Natl. Prod. Plant Resour. 2 (6), 649–664.

[B174] Prakash D. Gopinath K. Sudhandiran G. (2013). Fisetin enhances behavioral performances and attenuates reactive gliosis and inflammation during aluminum chloride-induced neurotoxicity. Neuromolecular Med. 15 (1), 192–208. 10.1007/s12017-012-8210-1 23315010

[B175] Prakash D. Sudhandiran G. (2015). Dietary flavonoid fisetin regulates aluminium chloride-induced neuronal apoptosis in cortex and hippocampus of mice brain. J. Nutr. Biochem. 26 (12), 1527–1539. 10.1016/j.jnutbio.2015.07.017 26411262

[B176] Prasath G. S. Subramanian S. P. (2013). Fisetin, a tetra hydroxy flavone recuperates antioxidant status and protects hepatocellular ultrastructure from hyperglycemia mediated oxidative stress in streptozotocin induced experimental diabetes in rats. Food Chem. Toxicol. 59, 249–255. 10.1016/j.fct.2013.05.062 23791753

[B177] Prasath G. S. Subramanian S. P. (2011). Modulatory effects of fisetin, a bioflavonoid, on hyperglycemia by attenuating the key enzymes of carbohydrate metabolism in hepatic and renal tissues in streptozotocin-induced diabetic rats. Eur. J. Pharmacol. 668 (3), 492–496. 10.1016/j.ejphar.2011.07.021 21816145

[B178] Prasath G. S. Sundaram C. S. Subramanian S. P. (2013). Fisetin averts oxidative stress in pancreatic tissues of streptozotocin-induced diabetic rats. Endocrine 44 (2), 359–368. 10.1007/s12020-012-9866-x 23277230

[B179] Rane A. R. Paithankar H. Hosur R. V. Choudhary S. (2021). Modulation of α-synuclein fibrillation by plant metabolites, daidzein, fisetin and scopoletin under physiological conditions. Int. J. Biol. Macromol. 182, 1278–1291. 10.1016/j.ijbiomac.2021.05.071 33991558

[B180] Reagan L. P. Magariños A. M. Yee D. K. Swzeda L. I. Van Bueren A. McCall A. L. (2000). Oxidative stress and HNE conjugation of GLUT3 are increased in the hippocampus of diabetic rats subjected to stress. Brain Res. 862 (1-2), 292–300. 10.1016/s0006-8993(00)02212-5 10799703

[B181] Reddy R. A. Benerji T. S. Shanker K. Prasad P. J. Perumal V. Kumar B. H. (2021). Fisetin, potential flavonoid with multifarious targets for treating neurological disorders: An updated review. Eur. J. Pharmacol. 910, 174492. 10.1016/j.ejphar.2021.174492 34516952

[B182] Rego A. C. Oliveira C. R. (2003). Mitochondrial dysfunction and reactive oxygen species in excitotoxicity and apoptosis: Implications for the pathogenesis of neurodegenerative diseases. Neurochem. Res. 28 (10), 1563–1574. 10.1023/a:1025682611389 14570402

[B183] Rein M. J. Renouf M. Cruz-Hernandez C. Actis-Goretta L. Thakkar S. K. da Silva Pinto M. (2013). Bioavailability of bioactive food compounds: A challenging journey to bioefficacy. Br. J. Clin. Pharmacol. 75 (3), 588–602. 10.1111/j.1365-2125.2012.04425.x 22897361 PMC3575927

[B184] Reitz C. Mayeux R. (2014). Alzheimer disease: Epidemiology, diagnostic criteria, risk factors and biomarkers. Biochem. Pharmacol. 88 (4), 640–651. 10.1016/j.bcp.2013.12.024 24398425 PMC3992261

[B185] Renoudet V. V. Costa-Mallen P. Hopkins E. (2012). A diet low in animal fat and rich in N-hexacosanol and fisetin is effective in reducing symptoms of Parkinson’s disease. J. Med. Food 15 (8), 758–761. 10.1089/jmf.2012.0060 22846082

[B186] Ribeiro M. Silva A. C. Rodrigues J. Naia L. Rego A. C. (2013). Oxidizing effects of exogenous stressors in Huntington’s disease knock-in striatal cells—Protective effect of cystamine and creatine. Toxicol. Sci. 136 (2), 487–499. 10.1093/toxsci/kft199 24008831

[B187] Rong X. Jiang L. Qu M. Hassan S. S. ul Liu Z. (2020). Enhancing therapeutic efficacy of donepezil by combined therapy: A comprehensive review. Curr. Pharm. Des. 27, 332–344. 10.2174/1381612826666201023144836 33100197

[B188] Saccon R. A. Bunton-Stasyshyn R. K. Fisher E. M. Fratta P. (2013). Is SOD1 loss of function involved in amyotrophic lateral sclerosis? Brain 136 (8), 2342–2358. 10.1093/brain/awt097 23687121 PMC3722346

[B189] Sagara Y. Vanhnasy J. Maher P. (2004). Induction of PC12 cell differentiation by flavonoids is dependent upon extracellular signal-regulated kinase activation. J. Neurochem. 90 (5), 1144–1155. 10.1111/j.1471-4159.2004.02563.x 15312169

[B190] Sakai E. Shimada-Sugawara M. Yamaguchi Y. Sakamoto H. Fumimoto R. Fukuma Y. (2013). Fisetin inhibits osteoclastogenesis through prevention of RANKL-induced ROS production by Nrf2-mediated up-regulation of phase II antioxidant enzymes. J. Pharmacol. Sci. 121 (4), 288–298. 10.1254/jphs.12243fp 23538677

[B191] Salganik R. I. (2001). The benefits and hazards of antioxidants: Controlling apoptosis and other protective mechanisms in cancer patients and the human population. J. Am. Coll. Nutr. 20 (5), 464S–472S. 10.1080/07315724.2001.10719185 11603657

[B192] Salim S. (2017). Oxidative stress and the central nervous system. J. Pharmacol. Exp. Ther. 360 (1), 201–205. 10.1124/jpet.116.237503 27754930 PMC5193071

[B193] Sari E. N. Soysal Y. Molecular and therapeutic effects of fisetin flavonoid in diseases. J. Basic Clin. Health Sci. 10.30621/jbachs.2020.1171

[B194] Sbardella E. Greco A. Stromillo M. L. Prosperini L. Puopolo M. Cefaro L. A. (2013). Isoprostanes in clinically isolated syndrome and early multiple sclerosis as biomarkers of tissue damage and predictors of clinical course. Mult. Scler. 19 (4), 411–417. 10.1177/1352458512457721 22917691

[B195] Schapira A. H. V. Chaudhuri K. R. Jenner P. (2017). Non-motor features of Parkinson disease. Nat. Rev. Neurosci. 18 (7), 435–450. 10.1038/nrn.2017.62 28592904

[B196] Sechi M. Syed D. N. Pala N. Mariani A. Marceddu S. Brunetti A. (2016). Nanoencapsulation of dietary flavonoid fisetin: Formulation and *in vitro* antioxidant and α-glucosidase inhibition activities. Mat. Sci. Eng. C Mat. Biol. Appl. 68, 594–602. 10.1016/j.msec.2016.06.042 27524059

[B197] Seet R. C. Lee C. Y. Lim E. C. Tan J. J. Quek A. M. Chong W. L. (2010). Oxidative damage in Parkinson disease: Measurement using accurate biomarkers. Free Radic. Biol. Med. 48 (4), 560–566. 10.1016/j.freeradbiomed.2009.11.026 19969070

[B198] Sengupta B. Banerjee A. Sengupta P. K. (2004). Investigations on the binding and antioxidant properties of the plant flavonoid fisetin in model biomembranes. FEBS Lett. 570 (1-3), 77–81. 10.1016/j.febslet.2004.06.027 15251443

[B199] Shams ul Hassan S. Ishaq M. Zhang W. Jin H.-Z. (2021). An overview of the mechanisms of marine fungi-derived antiinflammatory and anti-tumor agents and their novel role in drug targeting. Curr. Pharm. Des. 27, 2605–2614. 10.2174/1381612826666200728142244 32723250

[B200] Sheikh S. Safia H. E. Haque E. Mir S. S. (2013). Neurodegenerative diseases: Multifactorial conformational diseases and their therapeutic interventions. J. Neurodegener. Dis. 2013, 563481. Article ID 563481. 10.1155/2013/563481 26316993 PMC4437348

[B201] Shelat P. B. Chalimoniuk M. Wang J. H. Strosznajder J. B. Lee J. C. Sun A. Y. (2008). Amyloid beta peptide and NMDA induce ROS from NADPH oxidase and AA release from cytosolic phospholipase A2 in cortical neurons. J. Neurochem. 106 (1), 45–55. 10.1111/j.1471-4159.2008.05347.x 18346200

[B202] Shia C. S. Tsai S. Y. Kuo S. C. Hou Y. C. Chao P. D. L. (2009). Metabolism and pharmacokinetics of 3, 3', 4', 7-tetrahydroxyflavone (fisetin), 5-hydroxyflavone, and 7-hydroxyflavone and antihemolysis effects of fisetin and its serum metabolites. J. Agric. Food Chem. 57 (1), 83–89. 10.1021/jf802378q 19090755

[B203] Simonian N. A. Coyle J. T. (1996). Oxidative stress in neurodegenerative diseases. Annu. Rev. Pharmacol. Toxicol. 36 (1), 83–106. 10.1146/annurev.pa.36.040196.000503 8725383

[B204] Singh S. Singh A. K. Garg G. Rizvi S. I. (2018). Fisetin as a caloric restriction mimetic protects rat brain against aging induced oxidative stress, apoptosis and neurodegeneration. Life Sci. 193, 171–179. 10.1016/j.lfs.2017.11.004 29122553

[B205] Sinha R. Srivastava S. Joshi A. Joshi U. J. Govil G. (2014). *In-vitro* anti-proliferative and anti-oxidant activity of galangin, fisetin and quercetin: Role of localization and intermolecular interaction in model membrane. Eur. J. Med. Chem. 79, 102–109. 10.1016/j.ejmech.2014.04.002 24727463

[B206] Sivandzade F. Prasad S. Bhalerao A. Cucullo L. (2019). NRF2 and NF-қB interplay in cerebrovascular and neurodegenerative disorders: Molecular mechanisms and possible therapeutic approaches. Redox Biol. 21, 101059. 10.1016/j.redox.2018.11.017 PMC6302038 30576920

[B207] Smith R. G. Henry Y. K. Mattson M. P. Appel S. H. (1998). Presence of 4‐hydroxynonenal in cerebrospinal fluid of patients with sporadic amyotrophic lateral sclerosis. Ann. Neurol. 44 (4), 696–699. 10.1002/ana.410440419 9778272

[B208] Song K. Wu D. (2022). Shared decision-making in the management of patients with inflammatory bowel disease. World J. Gastroenterol. 28, 3092–3100. 10.3748/wjg.v28.i26.3092 36051346 PMC9331519

[B209] Sorolla M. A. Reverter-Branchat G. Tamarit J. Ferrer I. Ros J. Cabiscol E. (2008). Proteomic and oxidative stress analysis in human brain samples of Huntington disease. Free Radic. Biol. Med. 45 (5), 667–678. 10.1016/j.freeradbiomed.2008.05.014 18588971

[B210] Sowa M. Ślepokura K. Matczak-Jon E. (2013). Cocrystals of fisetin, luteolin and genistein with pyridinecarboxamide coformers: Crystal structures, analysis of intermolecular interactions, spectral and thermal characterization. CrystEngComm 15 (38), 7696–7708. 10.1039/c3ce41285g

[B211] Sowa M. Ślepokura K. Matczak-Jon E. (2014). Improving solubility of fisetin by cocrystallization. CrystEngComm 16 (46), 10592–10601. 10.1039/C4CE01713G

[B212] Spillantini M. G. Schmidt M. L. Lee V. M. Trojanowski J. Q. Jakes R. Goedert M. (1997). Alpha-synuclein in Lewy bodies. Nature 388 (6645), 839–840. 10.1038/42166 9278044

[B213] Strand A. D. Baquet Z. C. Aragaki A. K. Holmans P. Yang L. Cleren C. (2007). Expression profiling of Huntington’s disease models suggests that brain-derived neurotrophic factor depletion plays a major role in striatal degeneration. J. Neurosci. 27 (43), 11758–11768. 10.1523/JNEUROSCI.2461-07.2007 17959817 PMC6673215

[B214] Südhof T. C. Rizo J. (2011). Synaptic vesicle exocytosis. Cold Spring Harb. Perspect. Biol. 3 (12), a005637. 10.1101/cshperspect.a005637 22026965 PMC3225952

[B215] Syed D. N. Adhami V. M. Khan N. Khan M. I. Mukhtar H. (2016). Exploring the molecular targets of dietary flavonoid fisetin in cancer. Semin. Cancer Biol. 40-41, 130130–134040. 10.1016/j.semcancer.2016.04.003 PMC5067175 27163728

[B216] Sykiotis G. P. Habeos I. G. Samuelson A. V. Bohmann D. (2011). The role of the antioxidant and longevitypromoting Nrf2 pathway in metabolic regulation. Curr. Opin. Clin. Nutr. Metab. Care 14 (1), 41–48. 10.1097/MCO.0b013e32834136f2 21102319 PMC3092636

[B217] Tan S. Schubert D. Maher P. (2001). Oxytosis: A novel form of programmed cell death. Curr. Top. Med. Chem. 1 (6), 497–506. 10.2174/1568026013394741 11895126

[B218] Tordera M. Ferrándiz M. L. Alcaraz M. J. (1994). Influence of anti-inflammatory flavonoids on degranulation and arachidonic acid release in rat neutrophils. Z. Naturforsch. C J. Biosci. 49 (3-4), 235–240. 10.1515/znc-1994-3-412 8018254

[B219] Touil Y. S. Auzeil N. Boulinguez F. Saighi H. Regazzetti A. Scherman D. (2011). Fisetin disposition and metabolism in mice: Identification of geraldol as an active metabolite. Biochem. Pharmacol. 82 (11), 1731–1739. 10.1016/j.bcp.2011.07.097 21840301

[B220] Uddin M. S. Hossain M. F. Mamun A. A. Shah M. A. Hasana S. Bulbul I. J. (2020). Exploring the multimodal role of phytochemicals in the modulation of cellular signaling pathways to combat age-related neurodegeneration. Sci. Total Environ. 725, 138313. 10.1016/j.scitotenv.2020.138313 32464743

[B221] Ushikubo H. Watanabe S. Tanimoto Y. Abe K. Hiza A. Ogawa T. (2012). 3, 3', 4', 5, 5'-Pentahydroxyflavone is a potent inhibitor of amyloid β fibril formation. Neurosci. Lett. 513 (1), 51–56. 10.1016/j.neulet.2012.02.006 22343025

[B222] Van Houten B. Woshner V. Santos J. H. (2006). Role of mitochondrial DNA in toxic responses to oxidative stress. DNA Repair 5 (2), 145–152. 10.1016/j.dnarep.2005.03.002 15878696

[B223] Vielhaber S. Kunz D. WinKler K. Wiedemann F. R. KirchEs E. Feistner H. (2000). Mitochondrial DNA abnormalities in skeletal muscle of patients with sporadic amyotrophic lateral sclerosis. Brain 123, 1339–1348. 10.1093/brain/123.7.1339 10869047

[B224] Wang C-H. Liu H. Yang H. Wang F. (2015). Effects of fisetin on oxaliplatin-induced neuropathic pain in mice. Bangladesh J. Pharmacol. 10 (1), 138–142. 10.3329/bjp.v10i1.21203

[B225] Wang L. Tu Y. C. Lian T. W. Hung J. T. Yen J. H. Wu M. J. (2006). Distinctive antioxidant and anti-inflammatory effects of flavonols. J. Agric. Food Chem. 54 (26), 9798–9804. 10.1021/jf0620719 17177504

[B226] Wang T. Lin H. Tu Q. Liu J. Li X. (2016). Fisetin protects DNA against oxidative damage and its possible mechanism. Adv. Pharm. Bull. 6 (2), 267–270. 10.15171/apb.2016.037 27478791 PMC4961987

[B227] Wang T. H. Wang S. Y. Wang X. D. Jiang H. Q. Yang Y. Q. Wang Y. (2018). Fisetin exerts antioxidant and neuroprotective effects in multiple mutant h SOD1 models of amyotrophic lateral sclerosis by activating ERK. Neuroscience 379, 152–166. 10.1016/j.neuroscience.2018.03.008 29559385

[B228] Wang X.-H. Xu S. Zhou X.-Y. Zhao R. Lin Y. Cao J. (2021). Low chorionic villous succinate accumulation associates with recurrent spontaneous abortion risk. Nat. Commun. 12, 3428. 10.1038/s41467-021-23827-0 34103526 PMC8187647

[B229] Wang Y. Wang B. Lu J. Shi H. Gong S. Wang Y. (2017). Fisetin provides antidepressant effects by activating the tropomyosin receptor kinase B signal pathway in mice. J. Neurochem. 143 (5), 561–568. 10.1111/jnc.14226 28945929

[B230] Wang Y. X. Xia Z. H. Jiang X. Li L. X. An D. Wang H. G. (2019). Genistein inhibits aβ25-35-induced neuronal death with changes in the electrophysiological properties of voltage-gated sodium and potassium channels. Cell. Mol. Neurobiol. 39 (6), 809–822. 10.1007/s10571-019-00680-w 31037516 PMC11462837

[B231] Webber K. M. Raina A. K. Marlatt M. W. Zhu X. Prat M. I. Morelli L. (2005). The cell cycle in alzheimer disease: A unique target for neuropharmacology. Mech. Ageing Dev. 126 (10), 1019–1025. 10.1016/j.mad.2005.03.024 15936057

[B232] Whitney D. G. Shapiro D. N. Warschausky S. A. Hurvitz E. A. Peterson M. D. (2019). The contribution of neurologic disorders to the National prevalence of depression and anxiety problems among children and adolescents. Ann. Epidemiol. 29, 81–84. 10.1016/j.annepidem.2018.11.003 30545763 PMC6344250

[B233] Wu D. C. Teismann P. Tieu K. Vila M. Jackson-Lewis V. Ischiropoulos H. (2003). NADPH oxidase mediates oxidative stress in the 1-methyl-4-phenyl-1, 2, 3, 6-tetrahydropyridine model of Parkinson’s disease. Proc. Natl. Acad. Sci. U. S. A. 100 (10), 6145–6150. 10.1073/pnas.0937239100 12721370 PMC156340

[B234] Wu S.-J. Huang W.-C. Cheng C.-Y. Wang M.-C. Cheng S.-C. Liou C.-J. (2022). Fisetin suppresses the inflammatory response and oxidative stress in bronchial epithelial cells. Nutrients 14, 1841. 10.3390/nu14091841 35565807 PMC9103812

[B235] Wyss-Coray T. (2016). Ageing, neurodegeneration and brain rejuvenation. Nature 539 (7628), 180–186. 10.1038/nature20411 27830812 PMC5172605

[B236] Xi W. Wang X. Laue T. M. Denis C. L. (2016). Multiple discrete soluble aggregates influence polyglutamine toxicity in a Huntington’s disease model system. Sci. Rep. 6, 34916. 10.1038/srep34916 27721444 PMC5056504

[B237] Xiao S. Lu Y. Wu Q. Yang J. Chen J. Zhong S. (2021). Fisetin inhibits tau aggregation by interacting with the protein and preventing the formation of β-strands. Int. J. Biol. Macromol. 178, 381–393. 10.1016/j.ijbiomac.2021.02.210 33662414 PMC9022726

[B238] Xu M-X. Ge C. Li Q. Lou D. Hu L. Sun Y. (2020). Fisetin nanoparticles protect against PM2. 5 Exposure-induced neuroinflammation by down-regulation of astrocytes activation related NF-κB signaling pathway. J. Funct. Foods 65, 103716. 10.1016/j.jff.2019.103716

[B239] Yan J. Yao Y. Yan S. Gao R. Lu W. He W. (2020). Chiral protein supraparticles for tumor suppression and synergistic immunotherapy: An enabling strategy for bioactive supramolecular chirality construction. Nano Lett. 20, 5844–5852. 10.1021/acs.nanolett.0c01757 32589431

[B240] Yang W. Liu W. Li X. Yan J. He W. (2022). Turning chiral peptides into a racemic supraparticle to induce the self-degradation of MDM2. J. Adv. Res. 10.1016/j.jare.2022.05.009 PMC10006529 35667548

[B241] Yankner B. A. Lu T. Loerch P. (2008). The aging brain. Annu. Rev. Pathol. 3, 41–66. 10.1146/annurev.pathmechdis.2.010506.092044 18039130

[B242] Yao X. Li L. Kandhare A. D. Mukherjee-Kandhare A. A. Bodhankar S. L. (2020). Attenuation of reserpine-induced fibromyalgia via ROS and serotonergic pathway modulation by fisetin, a plant flavonoid polyphenol. Exp. Ther. Med. 19 (2), 1343–1355. 10.3892/etm.2019.8328 32010308 PMC6966137

[B243] Yoon W. B. Choi H. J. Kim J. E. Park J. W. Kang M. J. Bae S. J. (2018). Comparison of scopolamine-induced cognitive impairment responses in three different ICR stocks. Lab. Anim. Res. 34 (4), 317–328. 10.5625/lar.2018.34.4.317 30671121 PMC6333609

[B244] Yousefzadeh M. J. Zhu Y. McGowan S. J. Angelini L. Fuhrmann-Stroissnigg H. Xu M. (2018). Fisetin is a senotherapeutic that extends health and lifespan. EBiomedicine 36, 18–28. 10.1016/j.ebiom.2018.09.015 30279143 PMC6197652

[B245] Yu X. Jiang X. Zhang X. Chen Z. Xu L. Chen L. (2016). The effects of fisetin on lipopolysaccharide-induced depressive-like behavior in mice. Metab. Brain Dis. 31 (5), 1011–1021. 10.1007/s11011-016-9839-5 27209403

[B246] Zeng Q. Bie B. Guo Q. Yuan Y. Han Q. Han X. (2020). Hyperpolarized Xe NMR signal advancement by metal-organic framework entrapment in aqueous solution. Proc. Natl. Acad. Sci. U. S. A. 117, 17558–17563. 10.1073/pnas.2004121117 32661173 PMC7395552

[B247] Zhan J. Q. Chen C. N. Wu S. X. Wu H. J. Zou K. Xiong J. W. (2021). Flavonoid fisetin reverses impaired hippocampal synaptic plasticity and cognitive function by regulating the function of AMPARs in a male rat model of Schizophrenia. J. Neurochem. 158 (2), 413–428. 10.1111/jnc.15370 33882624

[B248] Zhang B. Y. Saijilafu Liu C. M. Wang R. Y. Zhu Q. Jiao Z. (2014). Akt-independent GSK3 inactivation downstream of PI3K signaling regulates mammalian axon regeneration. Biochem. Biophys. Res. Commun. 443 (2), 743–748. 10.1016/j.bbrc.2013.12.037 24333443 PMC3916952

[B249] Zhang H. Zheng W. Feng X. Yang F. Qin H. Wu S. (2019). Nrf2-ARE signaling acts as master pathway for the cellular antioxidant activity of fisetin. Molecules 24 (4), 708. 10.3390/molecules24040708 30781396 PMC6413105

[B250] Zhang L. Wang H. Zhou Y. Zhu Y. Fei M. (2018). Fisetin alleviates oxidative stress after traumatic brain injury via the Nrf2-ARE pathway. Neurochem. Int. 118, 304–313. 10.1016/j.neuint.2018.05.011 29792955

[B251] Zhang M. An C. Gao Y. Leak R. K. Chen J. Zhang F. (2013). Emerging roles of Nrf2 and phase II antioxidant enzymes in neuroprotection. Prog. Neurobiol. 100, 30–47. 10.1016/j.pneurobio.2012.09.003 23025925 PMC3623606

[B252] Zhang Q. Fujino M. Xu J. Li X. K. (2015). The role and potential therapeutic application of myeloid-derived suppressor cells in allo-and autoimmunity. Mediat. Inflamm. 2015, 421927. 10.1155/2015/421927 PMC4452474 26078493

[B253] Zhang X. Liu L. Chen W.-C. Wang F. Cheng Y.-R. Liu Y.-M. (2022). Gestational leucylation suppresses embryonic T-box transcription factor 5 signal and causes congenital heart disease. Adv. Sci. 9, e2201034. 10.1002/advs.202201034 PMC9130917 35320615

[B254] Zhang X. Qu Y.-Y. Liu L. Qiao Y.-N. Geng H.-R. Lin Y. (2021). Homocysteine inhibits pro-insulin receptor cleavage and causes insulin resistance via protein cysteine-homocysteinylation. Cell. Rep. 37, 109821. 10.1016/j.celrep.2021.109821 34644569

[B255] Zhao W. Varghese M. Yemul S. Pan Y. Cheng A. Marano P. (2011). Peroxisome proliferator activator receptor gamma coactivator-1alpha (PGC-1α) improves motor performance and survival in a mouse model of amyotrophic lateral sclerosis. Mol. Neurodegener. 6 (1), 51. 10.1186/1750-1326-6-51 21771318 PMC3156746

[B256] Zhao X. Li X. L. Liu X. Wang C. Zhou D. S. Ma Q. (2015). Antinociceptive effects of fisetin against diabetic neuropathic pain in mice: Engagement of antioxidant mechanisms and spinal GABAA receptors. Pharmacol. Res. 102, 286–297. 10.1016/j.phrs.2015.10.007 26520392

[B257] Zhao X. Wang C. Cui W. G. Ma Q. Zhou W. H. (2015). Fisetin exerts antihyperalgesic effect in a mouse model of neuropathic pain: Engagement of spinal serotonergic system. Sci. Rep. 5 (1), 9043. 10.1038/srep09043 25761874 PMC4356956

[B258] Zhen L. Zhu J. Zhao X. Huang W. An Y. Li S. (2012). The antidepressant-like effect of fisetin involves the serotonergic and noradrenergic system. Behav. Brain Res. 228 (2), 359–366. 10.1016/j.bbr.2011.12.017 22197297

[B259] Zheng L. T. Ock J. Kwon B. M. Suk K. (2008). Suppressive effects of flavonoid fisetin on lipopolysaccharide-induced microglial activation and neurotoxicity. Int. Immunopharmacol. 8 (3), 484–494. 10.1016/j.intimp.2007.12.012 18279803

[B260] Zhuo Z. Wan Y. Guan D. Ni S. Wang L. Zhang Z. (2020). A loop-based and AGO-incorporated virtual screening model targeting AGO-mediated miRNA-mRNA interactions for drug discovery to rescue bone phenotype in genetically modified mice. Adv. Sci. 7, 1903451. 10.1002/advs.201903451 PMC7341099 32670749

[B261] Zuo L. Shiah A. Roberts W. J. Chien M. T. Wagner P. D. Hogan M. C. (2013). Low Po₂ conditions induce reactive oxygen species formation during contractions in single skeletal muscle fibers. Am. J. Physiol. Regul. Integr. Comp. Physiol. 304 (11), R1009–R1016. 10.1152/ajpregu.00563.2012 23576612 PMC3680753

[B262] Zuo L. Zhou T. Pannell B. K. Ziegler A. C. Best T. M. (2015). Biological and physiological role of reactive oxygen species–the good, the bad and the ugly. Acta physiol. (Oxf). 214 (3), 329–348. 10.1111/apha.12515 25912260

[B263] Zweifel L. S. Parker J. G. Lobb C. J. Rainwater A. Wall V. Z. Fadok J. P. (2009). Disruption of NMDAR-dependent burst firing by dopamine neurons provides selective assessment of phasic dopamine-dependent behavior. Proc. Natl. Acad. Sci. U. S. A. 106, 7281–7288. 10.1073/pnas.0813415106 19342487 PMC2678650

