# Corrigendum: The neuroprotective effects of fisetin, a natural flavonoid in neurodegenerative diseases: Focus on the role of oxidative stress

**DOI:** 10.3389/fphar.2022.1095648

**Published:** 2022-11-23

**Authors:** Syed Shams ul Hassan, Saptadip Samanta, Raju Dash, Tomasz M. Karpiński, Emran Habibi, Abdul Sadiq, Amirhossin Ahmadi, Simona Bungau

**Affiliations:** ^1^ Shanghai Key Laboratory for Molecular Engineering of Chiral Drugs, School of Pharmacy, Shanghai Jiao Tong University, Shanghai, China; ^2^ Department of Natural Product Chemistry, School of Pharmacy, Shanghai Jiao Tong University, Shanghai, China; ^3^ Department of Physiology, Midnapore College, Midnapore, West Bengal, India; ^4^ Department of Anatomy, Dongguk University College of Medicine, Gyeongju, South Korea; ^5^ Department of Medical Microbiology, University of Medical Sciences, Poznań, Poland; ^6^ Department of Pharmacognosy, Faculty of Pharmacy, Mazandaran University of Medical Sciences, Sari, Iran; ^7^ Department of Pharmacy, University of Malakand, Chakdara, Pakistan; ^8^ Pharmaceutical Sciences Research Centre, Faculty of Pharmacy, Mazandaran University of Medical Sciences, Sari, Iran; ^9^ Department of Pharmacy, Faculty of Medicine and Pharmacy, University of Oradea, Oradea, Romania

**Keywords:** neurodegeneration, flavonoid, antioxidant, oxidative strees, fiestin

In the published article, an author name was incorrectly written as “Simona Bunagu”. The correct spelling is “Simona Bungau”.

The authors apologize for this error and state that this does not change the scientific conclusions of the article in any way. The original article has been updated.

