# Corrigendum: The neuroprotective effects of fisetin, a natural flavonoid in neurodegenerative diseases: focus on the role of oxidative stress

**DOI:** 10.3389/fphar.2024.1431842

**Published:** 2024-06-12

**Authors:** Syed Shams ul Hassan, Saptadip Samanta, Raju Dash, Tomasz M. Karpiński, Emran Habibi, Abdul Sadiq, Amirhossein Ahmadi, Simona Gabriela Bungau

**Affiliations:** ^1^ Shanghai Key Laboratory for Molecular Engineering of Chiral Drugs, School of Pharmacy, Shanghai Jiao Tong University, Shanghai, China; ^2^ Department of Natural Product Chemistry, School of Pharmacy, Shanghai Jiao Tong University, Shanghai, China; ^3^ Department of Physiology, Midnapore College, Midnapore, West Bengal, India; ^4^ Department of Anatomy, Dongguk University College of Medicine, Gyeongju, Republic of Korea; ^5^ Department of Medical Microbiology, Poznań University of Medical Sciences, Poznań, Poland; ^6^ Department of Pharmacognosy, Faculty of Pharmacy, Mazandaran University of Medical Sciences, Sari, Iran; ^7^ Department of Pharmacy, University of Malakand, Chakdara, Pakistan; ^8^ Pharmaceutical Sciences Research Centre, Faculty of Pharmacy, Mazandaran University of Medical Sciences, Sari, Iran; ^9^ Department of Pharmacy, Faculty of Medicine and Pharmacy, University of Oradea, Oradea, Romania

**Keywords:** neurodegeneration, flavonoid, antioxidant, fiestin, oxidative stress

In the published article, there was an error in the **Author contributions** statement. The statement was incomplete and did not communicate the precise contribution of each author. The correct **Author contributions** statement appears below.

